# Benefit of Insecticide-Treated Nets, Curtains and Screening on Vector Borne Diseases, Excluding Malaria: A Systematic Review and Meta-analysis

**DOI:** 10.1371/journal.pntd.0003228

**Published:** 2014-10-09

**Authors:** Anne L. Wilson, Ramesh C. Dhiman, Uriel Kitron, Thomas W. Scott, Henk van den Berg, Steven W. Lindsay

**Affiliations:** 1 School of Biological and Biomedical Sciences, Durham University, Stockton Road, Durham, United Kingdom; 2 National Institute of Malaria Research (ICMR), New Delhi, India; 3 Department of Environmental Sciences, Emory University, Atlanta, Georgia, United States of America; 4 Fogarty International Center, National Institutes of Health, Bethesda, Maryland, United States of America; 5 Department of Entomology and Nematology, University of California, Davis, California, United States of America; 6 Laboratory of Entomology, Wageningen University, Wageningen, Netherlands; Centers for Disease Control and Prevention, Puerto Rico, United States of America

## Abstract

**Introduction:**

Insecticide-treated nets (ITNs) are one of the main interventions used for malaria control. However, these nets may also be effective against other vector borne diseases (VBDs). We conducted a systematic review and meta-analysis to estimate the efficacy of ITNs, insecticide-treated curtains (ITCs) and insecticide-treated house screening (ITS) against Chagas disease, cutaneous and visceral leishmaniasis, dengue, human African trypanosomiasis, Japanese encephalitis, lymphatic filariasis and onchocerciasis.

**Methods:**

MEDLINE, EMBASE, LILACS and Tropical Disease Bulletin databases were searched using intervention, vector- and disease-specific search terms. Cluster or individually randomised controlled trials, non-randomised trials with pre- and post-intervention data and rotational design studies were included. Analysis assessed the efficacy of ITNs, ITCs or ITS versus no intervention. Meta-analysis of clinical data was performed and percentage reduction in vector density calculated.

**Results:**

Twenty-one studies were identified which met the inclusion criteria. Meta-analysis of clinical data could only be performed for four cutaneous leishmaniasis studies which together showed a protective efficacy of ITNs of 77% (95%CI: 39%–91%). Studies of ITC and ITS against cutaneous leishmaniasis also reported significant reductions in disease incidence. Single studies reported a high protective efficacy of ITS against dengue and ITNs against Japanese encephalitis. No studies of Chagas disease, human African trypanosomiasis or onchocerciasis were identified.

**Conclusion:**

There are likely to be considerable collateral benefits of ITN roll out on cutaneous leishmaniasis where this disease is co-endemic with malaria. Due to the low number of studies identified, issues with reporting of entomological outcomes, and few studies reporting clinical outcomes, it is difficult to make strong conclusions on the effect of ITNs, ITCs or ITS on other VBDs and therefore further studies be conducted. Nonetheless, it is clear that insecticide-treated materials such as ITNs have the potential to reduce pathogen transmission and morbidity from VBDs where vectors enter houses.

## Author Summary

Malaria is a deadly disease caused by a parasite which is transmitted by anopheline mosquitoes. Bednets treated with insecticide are one of the key tools used to prevent malaria and they have been distributed on a large scale in many countries, particularly in Africa. It may be possible to control other diseases transmitted by insects using insecticide-treated bednets because many of these insects also enter houses. We did a review of studies looking at the effectiveness of insecticide-treated bednets, curtains and house screening against nine major diseases transmitted by insects. We assessed the effect these tools had on reducing numbers of the insects and disease in humans. Insecticide-treated bednets were found to be effective in preventing cutaneous leishmaniasis—a disease transmitted by sandflies—and insecticide-treated curtains and screening showed potential in preventing other insect borne diseases. Although further studies are required, it is clear that insecticide-treated bednets, curtains and screening have the potential to prevent transmission of insect-transmitted diseases.

## Introduction

The World Health Organisation (WHO) promotes the use of Integrated Vector Management (IVM) to control vector borne diseases (VBDs) [1]. Briefly, IVM involves the use of a range of proven vector control tools used either alone or in combination selected based on knowledge of the local vector ecology and epidemiological situation. IVM can involve use of multiple vector control tools against a single disease or alternatively a single tool against multiple diseases. This is particularly the case where vector control interventions are active against more than one disease and VBDs overlap in their distribution.

IVM is a WHO policy for effective, cost effective and sustainable vector control. In order to exploit synergies between VBDs and make vector control more cost effective, IVM advocates for the use of shared interventions across diseases. However, in order to be able to do this it is important to first know whether interventions are effective against multiple diseases. This was the rationale for conducting this review. We considered insecticide-treated bednets (ITNs) since this intervention has been rolled out already on a large scale for malaria vector control.

ITNs form the mainstay of malaria vector control in many malaria endemic areas [2]. ITNs are estimated to reduce all-cause child mortality by 17% and uncomplicated *Plasmodium falciparum* episodes in areas of stable transmission by 50%, compared to no nets [3]. ITNs have been rolled out in malaria-endemic regions on a large scale, particularly in sub-Saharan Africa (SSA). Between 2004 and 2010, the number of ITNs delivered by manufacturers to malaria endemic countries in SSA increased from 6 million to 145 million [2]. The percentage of households owning at least one ITN in SSA is estimated to have risen from 3% in 2000 to 56% in 2012, but declined slightly to 54% in 2013. More work is needed to reach ITN coverage targets set by Roll Back Malaria of 80% use of ITNs by individuals in populations at risk [4]. Outside Africa, 60 million ITNs were distributed during 2009–2012, with 10 countries accounting for 75% of the total (India 9.2 million, Indonesia 6.1 million, Myanmar 5.4 million, Bangladesh 4.7 million, Afghanistan 4.3 million, Cambodia 3.6 million, Papua New Guinea 3.2 million, Haiti 3.0 million and Philippines 3.0 million) [2]. More recently conventional ITNs have been replaced by long lasting insecticide-treated nets (LLINs) that maintain effective levels of insecticide for at least three years meaning that re-treatment with insecticide is not necessary. Since 2007 the WHO recommends only use of LLINs and not conventional ITNs [5]. For the purpose of this review we refer to ITNs without distinguishing between conventional ITNs or LLINs.

ITNs are likely to be effective against multiple vectors and VBDs since a substantial proportion of transmission occurs indoors, but this has not been systematically assessed. As such there may be unknown collateral benefits of ITN roll-out on VBDs in addition to malaria. ITNs as well as insecticide-treated curtains (ITC) and insecticide-treated screening are likely to function in the same way. Disease vectors are attracted to host odours emanating either from people sleeping under ITNs or from people within houses in the case of ITCs and ITS. Vectors then coming into contact with these materials are deterred or killed and thus it can be said that the ITN and house are acting as ‘baited traps’. ITC and ITS may also be working to some extent to prevent vectors from entering houses (household level protection) rather than personal protection in the case of ITNs.

We conducted a systematic review to assess the efficacy of ITNs, ITCs or ITS against eight VBDs prioritised by the WHO in the Handbook for IVM [6]: Chagas disease, cutaneous and visceral leishmaniasis, dengue, human African trypanosomiasis, Japanese encephalitis, lymphatic filariasis and onchocerciasis. In this study we assessed the effect of ITNs, ITCs and ITS on clinical and entomological outcomes.

## Methods

### Literature search

The review was carried out according to a protocol and analytical plan that was prepared in advance. A systematic search of published literature was performed in April 2013 and repeated in June 2014 using intervention-specific search terms (for example ITN/LLIN/bednet/curtain/pyrethrins), as well as vector and disease specific search terms. MeSH and DeCS terms were used where appropriate. More detail on the search terms used is given in Supporting Information S1. MEDLINE (1950 -), EMBASE (1980 -) and LILACS (1982 -) databases were searched and no language restrictions were applied. In April 2013 we also searched the Tropical Disease Bulletin (1912 -) database. In addition, we reviewed the reference lists of key review articles and consulted with experts to identify further studies.

The search was conducted as part of a larger systematic review on *all* types of vector control interventions against eight different VBDs [6]: Chagas disease, cutaneous and visceral leishmaniasis, dengue, human African trypanosomiasis, Japanese encephalitis, lymphatic filariasis and onchocerciasis.

AW screened the search results for potentially relevant studies and full text documents were obtained for those publications deemed to be relevant. Foreign language studies were evaluated by a native speaker in consultation with AW. The articles were scrutinised to ensure that multiple publications from the same study were included only once.

### Study inclusion and exclusion criteria

Studies were assessed against inclusion and exclusion criteria by AW and SL independently. Studies were included if they compared the efficacy of ITNs, ITCs or ITS versus no intervention (control group) in disease endemic areas. Excluded studies and reasons for their exclusion are detailed in Supporting Information S2. We sought to compare the efficacy of ITNs, ITCs and ITS versus no intervention, rather than assess the efficacy of untreated bednets, curtains or screening or compare these untreated materials to ITNs, ITCs or ITS. We took this decision because bednets being rolled out for malaria control are insecticide-treated. Studies using hand-impregnated nets or factory manufactured LLINs were included. Studies assessed the effect of the intervention on either i) clinical outcomes (incidence or prevalence of disease or infection – whether this was confirmed by the patient, clinical diagnosis or diagnostically differed by study) and/or ii) entomological outcomes (including human biting rate, adult vector density and *Stegomyia* indices, pupal/demographic indices, oviposition rates or ovitrap positivity for dengue vectors). Adult vector density was measured using a number of techniques including Centers for Disease Control (CDC) light traps, sticky traps, pyrethrum spray catches and resting catches using aspirators. Larval indices extracted for dengue were house index (percentage of houses infested with larvae and/or pupae), container index (percentage of water containers infested with active immatures) and Breteau index (number of positive containers per 100 houses). We also extracted data on pupae per person (number of pupae collected over the total number of inhabitants of the households inspected), oviposition rates (mean number of *Aedes aegypti* eggs per trap) and ovitrap positivity (percentage of traps positive for *Aedes* eggs).

In terms of study designs, we included i) randomised controlled trials (cluster level or individual randomisation), ii) non-randomised trials with pre- and post-intervention data (for both control and intervention areas) and iii) rotational studies (provided there was baseline data or allocation was random or interventions/collectors were rotated appropriately e.g. each house received each intervention). A rotational design is when an intervention(s) is moved between sampling sites for set time periods or, in the case of human landing catches, collectors are rotated between interventions.

Studies performed under laboratory or semi-field conditions (for example, experimental huts) were excluded. We also excluded non-randomised trials without baseline data (for both control and intervention areas), non-controlled programme evaluations and observational studies in which clusters or individuals were not purposely allocated to intervention and control groups.

### Data extraction and analysis

AW (or a third party contractor) extracted data from the publications into a pre-designed data extraction form in Microsoft Word (Supporting Information S3), along with data tables and graphs. Graphs were digitised using Engauge Digitizer software (version 5.1, http://digitizer.sourceforge.net/). Preliminary analysis of data tables was conducted in Microsoft Excel. Analysis assessed the efficacy of ITNs, ITCs or ITS compared to no intervention. We used un-adjusted measures (clinical and entomological) throughout. This was for consistency because different studies adjust for different covariates. However, adjusted values, where available are reported for comparison.

Clinical outcomes were reported as either risks or rates of disease or infection in the published papers. Meta-analysis of clinical data (unadjusted risk of disease or infection) was performed in Stata 13 using the *metan* command (StataCorp, Texas, U.S.A.). Pre-intervention risk ratios were plotted on forest plots alongside post-intervention risk ratios to show comparability of groups at baseline. Statistical heterogeneity was assessed using a χ^2^ test. Due to the small number of studies in each comparison, we deemed there to be heterogeneity if the χ^2^ test p value was less than 0.1 [7]. If heterogeneity was found, a summary effect measure was calculated using random effect meta-analysis rather than fixed effect meta-analysis. Protective efficacy (PE) was calculated as PE = 1−(risk ratio of clinical disease or infection during the intervention period) ×100%. PE (or relative risk reduction) can be interpreted as the percentage reduction in risk of clinical disease or infection associated with the intervention. Standard formulas were used to calculate 95% confidence intervals for risk or rate ratios [8].

Entomological outcomes are reported as means with 95% confidence intervals, where these are reported in the published paper or could be calculated. If there were zero events then we estimated the upper 95% confidence interval as 100 x (3.7/N) where N is the sample size [9]. For entomological outcomes, where data were available for multiple intervention and control sites, we took the average values of the outcome measure, applying equal weight to all sites. A similar approach was taken if data were available for multiple timepoints within a year or transmission season, either pre- or post- intervention. We could not use meta-analysis to analyse the entomological data due to inadequate reporting in the published manuscripts. In almost all the studies the standard error for mean vector density was not reported and could not be calculated from the data presented in the papers. For studies with baseline/post intervention data for control and intervention sites we calculated the percentage reduction in vector density using a difference in differences approach. We estimated the effect of the intervention (J) using the formula J = (q1/q0)/(p1/p0), where q1 and q0 are, respectively, the entomological indicators (mean density, or biting rate) observed in the intervention and control areas post-intervention respectively and p1 and p0 are the corresponding baseline estimates of these entomological indicators [10]. We calculated the percentage reduction in entomological indicators as 100 x (1 - J). For studies in which only post intervention data were available we calculated the percent reduction in the outcome in the treatment group compared to the control group using the formula 100 x (1-(q1/q0) [10]. We were not able to calculate confidence intervals around percentage reductions due to heterogeneity in study designs; e.g. different follow up periods pre- and post-intervention and the way in which the data was reported e.g. the total vector count was reported rather than individual observations.

We followed recommendations made by the Preferred Reporting Items for Systematic Reviews and Meta-Analyses (PRISMA) group where possible [11], [12] (Supporting Information S4: PRISMA checklist).

### Risk of bias and study quality assessment

AW and SL assessed independently the risk of bias in the included studies using a risk of bias assessment form. This form was developed for the purposes of this review to assess entomological studies and was adapted from the Effective Practice and Organisation of Care (EPOC) risk of bias assessment form [13] (Supporting Information S5). A judgement of high, low or unclear risk of bias was given for a number of parameters. An overall bias assessment (high/medium/low) was made based on the modal bias risk.

We developed a tool for assessing study quality which primarily concerns the study design and downgrades the score given to the study depending on whether sample size calculations were performed (overall and for entomological sampling), the length of the follow up period and risk of bias (Supporting Information S6). This was loosely based on the Grading of Recommendations Assessment, Development and Evaluation (GRADE) system of rating quality of evidence [14], but adapted for entomological studies. For the purposes of the quality assessment, we deemed a trial to be a randomised controlled trial if the published paper stated that groups were randomised to intervention or control, even if the process of sequence generation was not described in the paper.

## Results

### Summary of studies identified and risk of bias and quality assessment

**Figure 1:**
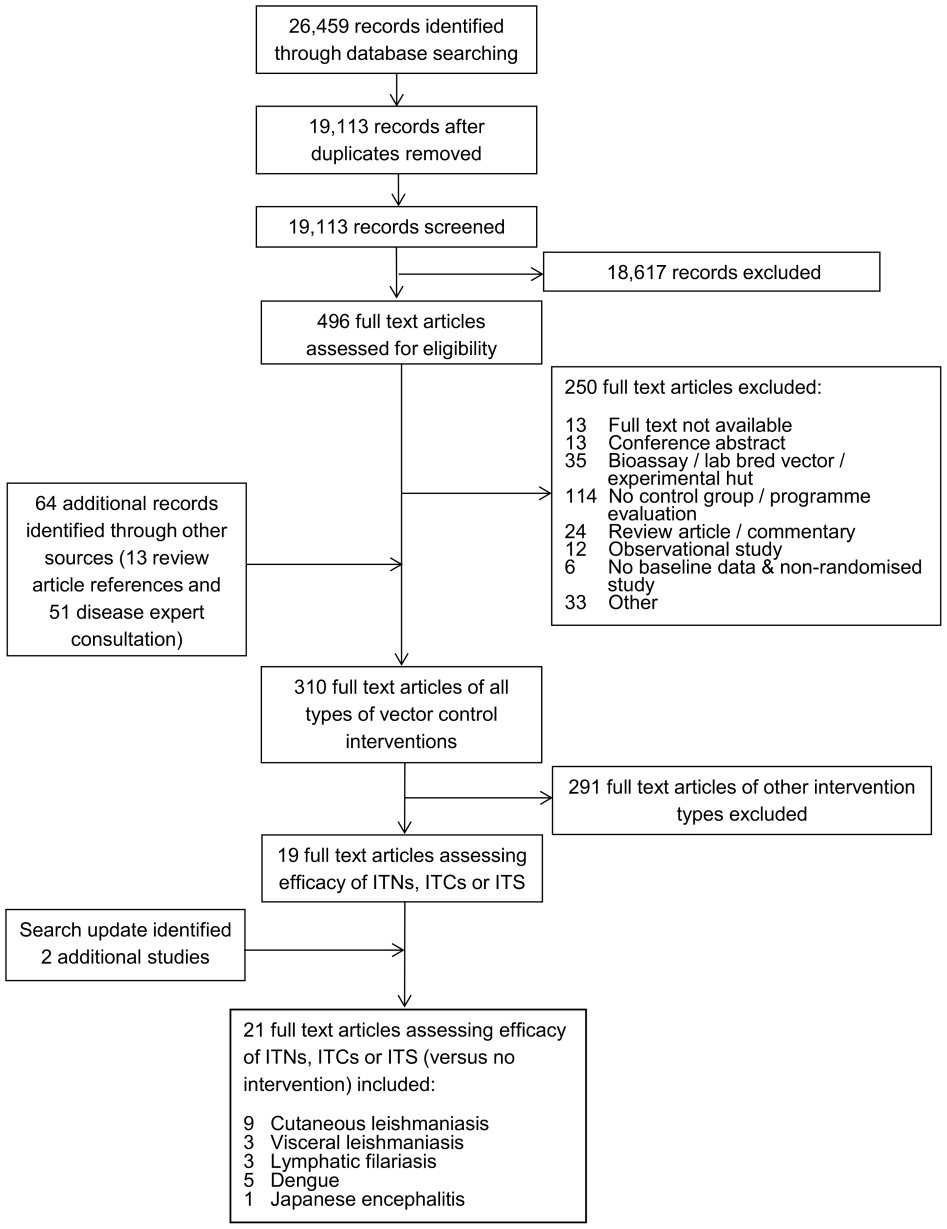
Flowchart of study inclusion (adapted from [11]).

The initial systematic literature search identified 19,113 unique records (Figure 1). 18,617 records were excluded based on review of the title and abstract. 496 full text records were reviewed and of these 310 studies met the inclusion/exclusion criteria across all types of vector control intervention. The update of the search in June 2014 identified 1,991 unique records, of which 125 full-text records were reviewed and 2 studies met the inclusion/exclusion criteria. In total, 21 studies assessed the efficacy of ITNs, ITCs or ITS versus no intervention and the split of these by disease was nine cutaneous leishmaniasis, five dengue, one Japanese encephalitis, three lymphatic filariasis and three visceral leishmaniasis. Summary tables of the studies identified are given in Supporting Information S7. Only nine of the 21 studies included reported the level of insecticide resistance in the study area or conducted an insecticide bioassay. Of the 21 studies identified, fifteen were deemed to be at low risk of bias, three at medium risk and three at high risk of bias [15] (Supporting Information S8). Twelve studies were deemed to be of high quality, three medium quality and six low quality (Supporting Information S9). No studies that met the inclusion and exclusion criteria were found assessing the efficacy of ITNs, ITCs or ITS against Chagas disease, human African trypanosomiasis or onchocerciasis.

### Efficacy of ITNs and ITCs against cutaneous leishmaniasis

A total of six studies assessing the efficacy of ITNs against cutaneous leishmaniasis were identified [15]–[20]. Of these three reported clinical data only, one reported entomological data only, and two reported both clinical and entomological data. Of the studies reporting clinical data, this was generally either a symptom questionnaire administered to participants or examination of lesions. Two studies utilised either a leishmanin skin test [20] or microscopic examination of skin scrapings from an active lesion [21].

**Figure 2:**
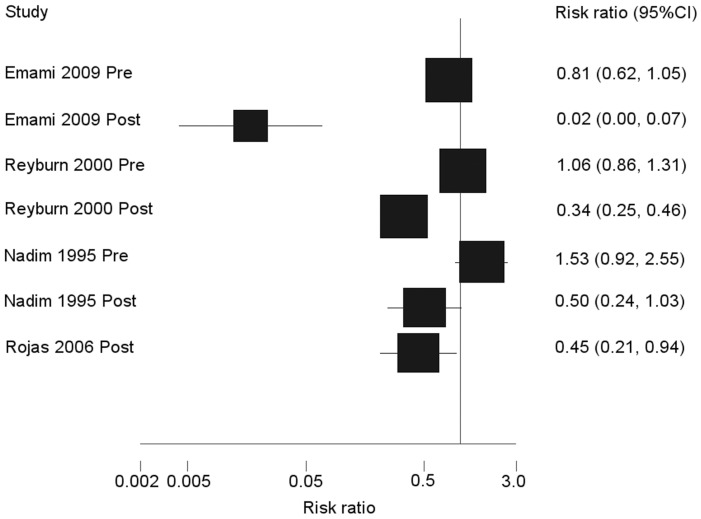
Forest plot (random effects meta-analysis) indicating efficacy of ITNs against cutaneous leishmaniasis. The forest plot displays post-intervention risk ratios and pre-intervention risk ratios separately to show comparability of groups at baseline.

Random effects meta-analysis of the efficacy of ITNs was conducted on data from four studies conducted in Iran (2 studies), Afghanistan and Colombia [17]–[20] (Figure 2, Table 1). Pre-intervention incidence of cutaneous leishmaniasis was comparable in intervention and control groups in the three studies that reported this data, with 95% confidence intervals for the risk ratio crossing the null value. Random effect meta-analysis indicated a PE of ITNs against cutaneous leishmaniasis of 77% (95% CI: 39%–91%, P = 0.003). Clinical data from one study in Turkey [15] was not suitable for meta-analysis because this study did not report numbers of cases or population at risk. Alten *et al*. reported a significant reduction in incidence of cutaneous leishmaniasis in ITN clusters, while incidence in control areas either stayed the same or increased. However, this study was deemed to be at high risk of bias and low quality.

**Table 1:** Effect of ITNs, ITCs and ITS against vector borne diseases.

Disease	Intervention	Study	Unadjusted PE (95% CI, p value)	Adjusted PE (95% CI, p value)	Covariates adjusted for
Cutaneous leishmaniasis	ITN	Emami 2009	98% (93%, 100%, p<0.001)	NR	NR
		Nadim 1995	50% (−3%, 76%, p = 0.06)	NR	NR
		Reyburn 2000	66% (54%, 75%, p<0.001)	69% (45%, 82%, p<0.001)	Intra-household clustering
		Rojas 2006	55% (6%, 79%, p = 0.03)	55% (−14%, 82%)	Age, residence located on the periphery, roof of thatch, distance to the forest <50 m, community participation score and prevalence of infection in children <5 years old
		Alten 2003	37%	NR	NR
	ITC	Kroeger 2002	93% (−16%, 100%, p = 0.06)	NR	NR
	ITC and ITS	Noazin 2013*	16% (2%, 28%, p = 0.03)	NR	NR
Visceral leishmaniasis	ITN	Picado 2010	Cases: 4% (−81%, 48%, p = 0.9) Infection: 0.3% (−15%, 14%, p = 0.97)	Cases: −15% (−116%, 39%, p = 0.64) Infection: 11% (−64%, 52%, p = 0.68)	Clustering, age group, sex, times sprayed, and socioeconomic status.
Dengue	ITS	Nguyen 1996 Igarashi 1997	81% (53%, 92%, p<0.001)	NR	NR
Japanese encephalitis	ITN	Dutta 2011	67% (44%, 80%, p<0.001)	NR	NR

*study reported rates only, PE  =  protective efficacy, CI  =  confidence interval, NR  =  not recorded, More detail on cases and denominators given in Supporting Information S10.

Studies assessing the efficacy of ITNs reported mixed results in terms of effect on sandfly density ranging from a relative *increase* of 49% to a relative reduction of 96% (Table 2). Although Emami *et al*. reported a highly significant PE against cutaneous leishmaniasis in Iran, no effect on the mean number of *Phlebotomus sergenti* captured per month was detected in this study [17]. Similarly, Alten *et al*. reported a beneficial effect of ITNs on clinical disease in Turkey and a percentage increase in vector density relative to the control group was documented [15].

**Table 2:** Effect of ITNs and ITCs on density of sandfly vectors of cutaneous leishmaniasis.

Study	Vector species	Surveillance method	Measure	Control (Mean and 95% CI)		Intervention (Mean and 95% CI)		% reduction in vector density	Adjusted % reduction	Covariates adjusted for
				Pre	Post	Pre	Post		(95% CI, p value)	
**ITNs**										
Alexander 1995	*Lutzomyia lichyi*, *L. youngi, L. columbiana*	HLC	No. of sandflies caught per man hour (biting inside net)		3.29		0.14	**96%**	NR	NR
Alten 2003	*Phlebotomus papatasi, P. sergenti*	CDC LT and sticky trap	Mean no of female *P. papatasi* and *P. sergenti*/month		88.8		132.2	**−49%**	NR	NR
Emami 2009	*P. papatasi, P. sergenti*	(exophilic and endophilic species, respectively)	Mean no of female *P. sergenti* captured/month	615	214	385	140	**−5%**	NR	NR
**ITCs**										
Alexander 1995	*L. lichyi, L. youngi, L. columbiana*	HLC	No. of sandflies caught per man hour (biting)		3.29		1.5	**54%**	NR	NR
Kroeger 2002	*L. youngi, L. ovallesi*	CDC LT	Mean no of sandflies per trap	19.5	20.0	15.1	2.1	**87%**	NR	NR
Majori 1989	*P. duboscqi*	PSC	Density of sandflies per single PSC	47	71	40	1	**98%**	NR	NR

where CDC LT  =  Centers for Disease Control light traps, HLC  =  human landing catches, PSC  =  pyrethrum spray catches and NR  =  not reported.

More detail on vector numbers and denominators given in Supporting Information S11.

Three studies conducted in Colombia, Venezuela and Burkina Faso assessed the efficacy of ITCs against cutaneous leishmaniasis [16], [22], [23]. Two studies reported entomological data while one reported both clinical and entomological data. Kroeger *et al.* demonstrated a high PE against cutaneous leishmaniasis of 93% (95% CI: −16%–100%, p = 0.06) in Venezuela (Table 1) [22]. Studies that measured the entomological effect of ITCs demonstrated a high percentage reduction in vector density of 54%, 87% and 98% (Table 2). However, the 98% reduction was observed in a study that was deemed to be of low quality due to the study design employed (non-randomised pre-post design), few sampling sites for entomological data and short period of follow up.

A study which assessed the efficacy of ITCs and ITS against cutaneous leishmaniasis in Iran reported a PE of 16% (95% CI: 2%–28%, p = 0.03) [21]. This study was deemed to be of low quality due to the study design (non-randomised pre-post design) and high risk of bias.

### Efficacy of ITNs against visceral leishmaniasis

Three studies assessing the efficacy of ITNs on visceral leishmaniasis were identified [24]–[27]. Two studies reported only entomological data and one reported both clinical and entomological data. The Picado *et al*. study [27] did not show a significant effect on incident *Leishmania donovani* infections (PE: 0.3%, 95%CI: −15%–14%, p = 0.97) or incident cases of visceral leishmaniasis (PE: 4%, 95%CI: −81%–48%, p = 0.9) in India and Nepal (Table 1). The same study, however, did appear to show an effect on vector density with a relative reduction in the mean number of *P. argentipes* females per light trap night of 57% [26] (Table 3). Two studies conducted in Sudan [24] and Bangladesh, India and Nepal [25] demonstrated a 100% and 35% (95% CI: −56% to 75%) reduction in vector density, respectively (Table 3).

**Table 3:** Effect of ITNs on density of sandfly vectors of visceral leishmaniasis.

Study	Vector species	Surveillance method	Measure	Control (Mean and 95% CI)		Intervention (Mean and 95% CI)		% reduction in vector density	Adjusted % reduction	Covariates adjusted for
				Pre	Post	Pre	Post		(95% CI, p value)	
**ITNs**										
Elnaiem 1999	*Phlebotomus orientalis*	HLC	Mean number of *P. orientalis* females landing to bite per volunteer/per night		32 (15.7, 48.3)		0 (0, 31)	**100%**	NR	NR
Joshi 2009	*P. argentipes, P. papatasi*	CDC LT	Number of sandflies per house (trap) per night (unadjusted mean)	9.41 (6.97, 12.71)	12.15 (8.68, 17)	9.92 (7.28, 13.53)	8.32 (5.56, 12.45)	**35%** *	Model coefficient reported only (−0.42, p = 0.04)	Clustering (country/cluster), type of wall and dwelling
Picado 2010	*P. argentipes*	CDC LT	Mean number of female *P. argentipes* per LT night	2.62	1.57	2.18	0.55	**58%**	11.6% (95%CI 2.10−20.2%), p = 0.016	Clustering (country/cluster), baseline mean density, IRS carried out by ministry in some clusters

where CDC LT  =  Centers for Disease Control light traps, HLC  =  human landing catches and NR  =  not reported.

* paper reports unadjusted PE as 43.7% (reduction in count = −4.34, 95% CI: −8.57, −0.10 and model coefficient: −0.43, p = 0.04)

More detail on vector numbers and denominators given in Supporting Information S11.

No studies were identified which assessed the efficacy of ITCs or ITS against visceral leishmaniasis.

### Efficacy of ITNs and ITCs against lymphatic filariasis

Two studies assessing the efficacy of ITNs against lymphatic filariasis were identified, both of which collected entomological data only [28], [29]. ITNs generally were associated with a high level of protection against *Anopheles* species, with approximately a 98% reduction in vector density in the two studies conducted in Kenya and Papua New Guinea (Table 4). Bøgh *et al*. reported a lower percentage reduction in *Culex quinquefasciatus* density of 16% [28].

**Table 4:** Effect of ITNs and ITCs on lymphatic filariasis vectors.

Study	Vector species	Surveillance method	Measure	Control (Mean and 95% CI)		Intervention (Mean and 95% CI)		% reduction in vector density	Adjusted % reduction	Covariates adjusted for
				Pre	Post	Pre	Post		(95% CI, p value)	
**ITNs**										
Bøgh 1998	*Anopheles gambiae s.l.*	PSC	Geometric mean no. of indoor resting female mosquitoes per house	29.7	12.3	17	0.1	**99%**	NR	NR
	*An. funestus*			20.4	33.4	19.7	0.7	**98%**	NR	NR
	*Culex quinquefasciatus*			14.6	5.0	7.7	2.2	**17%**	NR	NR
Charlwood 1987	*An. punctulatus*	Aspirator	No. of indoor resting *An. punctulatus* females	-	67.7	-	2	**97%**	NR	NR
**ITCs**										
Poopathi 1995	*Cx. quinquefasciatus*	HLC	Average man biting (landing) density per man hour	133.5	62.8	91.5	7.5	**83%**	NR	NR
		Aspirator	Average indoor resting density per man hour	55.0	55.9	42.5	9.0	**79%**	NR	NR

where HLC  =  human landing catches and PSC  =  pyrethrum spray catches.

More detail on vector numbers and denominators given in Supporting Information S11.

One study conducted in India assessing the efficacy of ITCs hung in eaves and doorways against lymphatic filariasis vectors was identified [30]. Poopathi *et al*. detected an 82% reduction in man biting density and a 79% reduction in indoor resting density of *Cx. quinquefasciatus* [30] (Table 3). However, this study was deemed to be of low quality mainly due to the study design employed (non-randomised pre-post design), few sampling sites for entomological data and short period of follow up.

### Efficacy of ITNs, ITCs and ITS against dengue

One study conducted in Haiti assessed the efficacy of ITNs against dengue [31]. Based on the five month post-intervention survey this study showed that ITN use was associated with a 36% reduction in pupae per person and 77% reduction in indoor ovitrap positivity. However, the study reported that ITNs were associated with a 56% increase in house index, 143% increase in container index, 60% increase in Breteau index and 20% increase in outdoor ovitrap positivity. The bioassay results on new nets from this study site indicated only 30% mortality of *A. aegypti* suggesting that insecticide resistance may have been a problem.

Three studies were identified that assessed the efficacy of ITCs against dengue vectors [32]–[34]. Kroeger *et al*. demonstrated in Mexico a beneficial effect of ITCs on house index (25% reduction) and pupae per person (39% reduction), but reported a relative *increase* in Breteau index of 10% based on the 12 month follow up survey [32]. The authors, however, reported a community-level effect of the ITCs which meant that benefits in terms of reductions in mosquito populations spilt over into control areas. They postulate that this is why there is no significant difference between intervention and control arms. Breteau and house indices from an external control area closely follow seasonal rainfall patterns and do not show similar reductions as in the study intervention and control areas. In Thailand Lenhart *et al*. did not detect a beneficial effect of ITCs on house index, container index, Breteau index or pupae per person, with relative *increases* of 15%, 20%, 3% and 37%, respectively at the nine-month time point [33]. ITCs did, however, show a beneficial effect on indoor and outdoor oviposition rates with reductions of 44% and 49% in mean numbers of eggs per trap, respectively at the six month time point, although no significant difference between control and ITC arms was reported at three or nine months. Another study in Thailand where houses generally had a more closed design reported a 56% reduction in house index, 67% reduction in Breteau index and 63% reduction in pupae per person index six months after the start of the intervention [34]. At the 6-month follow up survey 71% of households had at least one ITC. However, at the 18-month follow up survey when ITC coverage had fallen to only 33% a much lower effect on entomological parameters was observed (26% reduction in house index, 8% reduction in Breteau index and 111% increase in pupae per person index).

A study of ITS reported a beneficial effect on both house index and density index (adult *Ae. aegypti*) in Vietnam. In the intervention arm both house and density index were reduced to zero one month after installation of the screening and remained at zero for the duration of the epidemic season (eight months post intervention), compared to the control arm in which seasonal peaks in both indices were observed [35], [36]. The same study also reported a PE of ITS against IgM seropositivity of 80% (95% CI: 53–92%, p<0.001) compared to the control group (Table 1). This study used a non-randomised pre-post design and was deemed to be of low quality.

### Efficacy of ITNs against Japanese encephalitis

A single study by Dutta *et al*. assessed the efficacy of ITNs against Japanese encephalitis vectors and seroconversion in India [37]. This study was deemed to be of low quality due to the study design employed (non-randomised pre-post design) and low number of sampling sites for entomological data. No effect of ITNs on mean density of adults of the *Cx. vishnui* group was observed (reduction of −3.5%). The risk of seroconversion against Japanese encephalitis virus was comparable across groups at baseline, but the risk was significantly lower in the ITN group compared to the control during the two year post intervention period (PE: 67%, 95%CI: 44–80%, p<0.001) (Table 1).

## Discussion

Our review shows the potential for ITNs, ITCs and ITS to reduce vector borne diseases. Of particular note is the evidence on high protective efficacy of ITNs against cutaneous leishmaniasis, which suggests that there may be considerable collateral benefits of ITN roll out where cutaneous leishmaniasis and malaria are co-endemic. There is also good evidence of the efficacy of ITC and ITS against cutaneous leishmaniasis. Weaker evidence exists for the effect of ITS on dengue and ITNs on Japanese encephalitis, but these interventions look promising. Further studies should be conducted to confirm these findings. The potential of ITNs, ITCs and ITS against Chagas disease, human African trypanosomiasis and onchocerciasis remains untested. In several studies the pattern of reduction in disease incidence was not matched by reductions in entomological parameters. This is not unsurprising given the complicated relationship between vector density and risk of human infection, particularly when vector infection rate is not taken into account.

Meta-analysis showed that ITNs were able to reduce the incidence of cutaneous leishmaniasis by 77%. This finding provides support for WHO's recommendation that ITNs should be used as a vector control method against this disease [38]. This level of protective efficacy compares favourably with the 50% protective efficacy of ITNs against *P. falciparum* malaria shown by Lengeler [3]. Based on maps of cutaneous leishmaniasis [39] and *P. falciparum* endemicity [40] there are large areas, particularly in South America, where these diseases are likely to be co-endemic. Non-malaria endemic countries where cutaneous leishmaniasis is prevalent should consider rolling out ITNs as part of control efforts. Similar reductions in vector density were not observed which may be due to the ecology of the vector species or differences in collection techniques. For example studies by Alten *et al*. and Emami *et al*. sampled both endophilic and exophilic species [15], [17]. Studies by Kroeger *et al*. [22] and Noazin *et al*. [21] reported significant effects of ITC and ITC/ITS on clinical outcomes.

Clinical evidence from one study suggested that ITNs were not effective against visceral leishmaniasis [27]. However, in this study Picado *et al*. suggested that *L. donovani* transmission may have been occurring outside the home where ITNs would have little impact on preventing sandfly-human contact. In Africa observational studies led to mixed results – several studies have shown treated bednets to be protective against visceral leishmaniasis [41], [42], while others have shown no effect of ITNs on *L. donovani* infection rate in *P. orientalis*, although the number of infected *P. orientalis* identified was small in all villages [43]. In south Asia, several observational studies have shown use of (untreated) bednets to be protective against visceral leishmaniasis [44], [45].

The efficacy of ITNs in preventing leishmaniasis transmission is dependent on a number of key variables related to vector biology, type of nets and human behaviour. Studies have shown protection is dependent on mesh size of the nets – nets designed to be cooler which have large holes are more likely to let sandflies though, even if they are insecticide treated [46]. ITNs are likely to be more effective where sandflies bite indoors at night and where people use ITNs consistently [47], [48]. ITCs and ITS may be advantageous over ITNs because these interventions are in place all the time and since there is no need to set them up at night compliance is less of an issue [21]. In general, where transmission is occurring inside the home or where vectors rest indoors, we would expect ITNs, ITCs or ITS to have a beneficial effect, irrespective of whether the vectors are transmitting cutaneous or visceral leishmaniasis. It is important to have a sound grasp of sandfly biology and human behaviour in a particular setting in order to understand where transmission is occurring or where vectors rest before planning specific intervention strategies.

There were no studies that met the selection criteria, which reported the efficacy of ITNs against lymphatic filariasis infection. In much of SSA and parts of the western Pacific, *Anopheles* mosquitoes transmit both lymphatic filariasis and malaria and so theoretically ITNs should have a beneficial effect on both diseases [49]. Observational studies have shown a beneficial effect of ITNs on lymphatic filariasis transmission where the disease is transmitted by *Anopheles* mosquitoes [50]–[53] and ITNs may be particularly useful in areas co-endemic for lymphatic filariasis and *Loa Loa* where mass drug administration of ivermectin is contraindicated due to serious adverse events [54]. However, to our knowledge no randomised controlled trials have been performed in these settings. Such a study would need to be of long duration to show a reduction in microfilaraemia given that adult worms have lifespans of between four and 10 years [55], [56]. Alternatively, a study could use incidence of new infections in young children as an outcome [57]. The efficacy of ITNs, ITCs and ITS against *Culex* vectors of lymphatic filariasis, which are predominant in urban areas [58], needs further assessment. Bøgh *et al*. reported a 16% reduction in indoor resting density of *Cx. quinquefasciatus* compared to a 98% reduction in *Anopheles* species [28], presumably because *Culex* are less susceptible than *Anopheles* to pyrethroids [59]–[61]. Another explanation may be that transient reductions in vector density are masked because *Culex* populations are massive and the population can rapidly replace itself or immigrate. Poopathi *et al*. assessed the effect of insecticide-treated eave and door curtains and reported an 82% reduction in human biting density of *Cx. quinquefasciatus* [30]. It may be the door curtain component of this intervention which is of greatest importance given the findings of a study by Njie *et al*. who reported that culicines enter houses via the door rather than the eaves [62].

There is an increasing focus on intradomicile vector control for dengue [63] because *Ae. aegypti* rest, feed, mate and reproduce inside houses [64]. Targeting adult *Ae. aegypti* shifts the age structure of the vector population to younger mosquitoes, which is likely to have a large effect on human infections due to the relatively long extrinsic incubation period of the dengue virus in the mosquito [65]. However, since transmission of dengue occurs mostly during the daytime the use of bednets has rarely been considered as an intra-domiciliary control strategy. Studies identified in this review reporting an effect of ITCs and ITS on *Ae. aegypti* infestation levels [32], [34]–[36] suggest that vectors are coming into contact with these interventions indoors. The likelihood of the vector coming into contact with the ITN, ITC or ITS will depend on a number of factors including the size of the home and construction. For example, Lenhart *et al*. state that the open construction of the homes in their study conducted in Thailand may explain why ITCs did not show any effect [33]. It is generally recognised that greater coverage of the intervention will result in mass killing, reduced vector survival and greater reductions in transmission; i.e. a community level effect. This was apparent in two of the dengue studies included in this review. In one study use of ITCs in intervention areas led to a community level effect whereby larval indices were reduced in neighbouring control areas [32]. A study by Vanlerberghe reported that a reduction in ITC coverage over time led to a reduced effect on entomological parameters [34]. A similar pattern of coverage dependent effects of ITCs on *Ae. aegypti* larval and pupal/demographic indices was reported in another study in Venezuela, which suggested that at least 50% coverage of ITCs was necessary to reduce *Ae. aegypti* infestation levels by 50% [66].

Entomological data from studies on the efficacy of ITCs and ITNs against the dengue vector *Ae. aegypti* were inconsistent across the different indices measured. Focks and others have questioned the reliability and sensitivity of traditional immature *aegypti* indices (the house, container, and Breteau indices) and there is growing consensus that these indices are of little value in predicting risk of human infection [67]. Ovitraps are also not recommended for assessing vector abundance because measures are often biased by competition from natural oviposition sites [63]. Instead pupal/demographic indices (for example pupae per person) are a better proxy for adult vector abundance or measurement of adult vector density itself [67], [68] and are more appropriate for assessing transmission risk and directing control operations [69], [70]. The ideal would be to have a measure similar to the entomological inoculation rate for malaria transmission (incorporating both adult density and infection rate). However, adult *Ae. aegypti* are difficult to catch in appreciable numbers (though this is likely to improve with development of new adult monitoring tools) and only small proportion of adults are infected so it is difficult to detect infection [71].

The absence of studies of the two human trypanosome vectors and black flies is noteworthy. For black flies and tsetse flies, the predominantly outdoor exposure may be the main underlying reason. For triatomines, the absence of intensive bednet campaigns in Chagas disease endemic areas (which are often non-malarious, especially for the main vector *Triatoma infestans*), and the general lack of attention to improved housing may be among the principal underlying factors for the lack of studies.

Our review has several limitations that should be noted. We focused on a number of important neglected tropical diseases. This group of diseases is well-named because few studies were identified, despite conducting a comprehensive database search and contacting disease experts. We also relaxed the inclusion criteria somewhat in terms of study designs to include non-randomised studies with pre- and post-intervention data. We did not, however, do a full search of the grey literature which may mean that publication bias was introduced resulting in over-reporting of studies demonstrating that ITNs, ITCs and ITS were protective. We did not request further information from authors if reporting of methods or results was unclear in the published paper. Due to the few studies identified, summary estimates could only be generated using meta-analysis for cutaneous leishmaniasis. Studies were generally at low risk of bias but were of mixed quality. The main problems identified were with study design; e.g. short periods of follow up and incomplete reporting in the published papers; e.g. the method of sequence generation for randomisation was not reported. We took a cautious approach and did not calculate confidence intervals for entomological outcomes. This was due to i) heterogeneity in study designs e.g. differences in follow up periods pre- and post-intervention and between studies, studies involving single houses and entomological parameters measured once versus studies with multiple clusters and measurements over an extended time period and ii) incomplete reporting in the published papers e.g. confidence intervals and standard deviations omitted. Without knowing the uncertainty around percentage reductions it was not possible to make any conclusions regarding the entomological effect of interventions. Improved reporting of entomological data in studies and standardisation of study design and conduct should be a priority. Entomological data should always be assessed in combination with a clinical outcome where possible, and clinical outcomes with standardised diagnostic techniques and case definitions should remain the gold standard outcome for assessing the efficacy of vector control interventions.

Less than half of the studies we considered reported the results of bioassays for efficacy of the insecticide used. In one of the studies conducted in Haiti there was some indication of resistance [31]. However, many of the studies were conducted prior to the early 2000s before the advent of pyrethroid resistance [72], including those against cutaneous leishmaniasis that show a high PE. It is not possible, therefore, to say whether this level of efficacy would be observed today. Currently pyrethroids are the only class of insecticides suitable for use on LLINs and increasing coverage of pyrethroid treated materials to control multiple VBD is likely to increase selection pressure for development of resistance. Indeed, pyrethroid resistance has been detected in a number of non-malaria vectors including *Cx. quinquefasciatus* [73]–[75], sand flies [76], *Ae. aegypti* and *Ae. albopictus* [77]. Even if pyrethroid resistance increases it is likely that ITNs, ITC and ITS will still afford some level of protection against vectors due to a barrier effect. However, it would be sound to use insecticide treated materials as part of an IVM strategy including other vector control tools that do not rely on insecticide such as larval source management or make sure that different insecticide classes are used for IRS/fogging etc (if appropriate). In the meantime, new types of insecticide treated materials, for example LLINs impregnated with insecticides with two different modes of action, are being developed which are showing promise against insecticide resistant malaria vectors [78], [79].

In terms of collateral benefits there may also be beneficial effects of ITNs, curtains and screening on preventing household pests such as bedbugs, headlice, cockroaches and rodents which although not systematically assessed in this review are important benefits which increase acceptability and encourage compliance with interventions [80]–[82].

In conclusion, ITNs, ITCs and ITS have great potential to reduce VBDs. The biological insight that follows from this conclusion is that a substantial proportion of the vector population must be resting or feeding indoors. Evidence on efficacy of ITNs, ITC and ITS against multiple VBDs should be paired with maps of disease co-endemicity in order to prioritise and focus resources to areas of greatest disease burden. The use of interventions against multiple diseases has the potential to reduce costs and make better use of financial and human resources. This requires functional coordination between disease-specific programmes on planning, implementation and monitoring and evaluation with sharing of existing infrastructure and competencies. Beneficial effects on multiple VBDs will serve to increase the cost effectiveness of insecticide-treated materials and this may help to bolster the case for vector control funding. This review demonstrates some promising results, but highlights the urgent need for further well conducted studies. The efficacy of ITNs, ITCs and ITS against VBDs needs to be rigorously tested in randomised controlled trials with standardised clinical outcomes.

## Supporting Information

Supporting Information S1 Search terms.
(DOCX)

Supporting Information S2 Excluded studies and reasons for exclusion.
(DOCX)

Supporting Information S3 Data extraction form.
(DOCX)

Supporting Information S4 PRISMA checklist.
(DOCX)

Supporting Information S5 Risk of bias assessment form.
(DOCX)

Supporting Information S6 Study quality assessment form.
(DOCX)

Supporting Information S7 Characteristics of studies identified.
(DOCX)

Supporting Information S8 Assessment of risk of bias.
(DOCX)

Supporting Information S9 Assessment of study quality.
(DOCX)

Supporting Information S10 Detail of results presented in Table 1.
(XLSX)

Supporting Information S11 Detail of results presented in Tables 2, 3 and 4.
(XLSX)
