# Supplementary material for: Benefit of Insecticide-Treated Nets, Curtains and Screening on Vector Borne Diseases, Excluding Malaria: A Systematic Review and Meta-analysis
Source: PLoS Negl Trop Dis. 2014 Oct 9;8(10):e3228. doi: 10.1371/journal.pntd.0003228 (PMC4191944; doi:10.1371/journal.pntd.0003228)
Supplement: Supporting Information S1 — Search terms. (DOCX) [file pntd.0003228.s001.docx]

**Supporting information S1: Search terms**

| **Search set** | **Medline** | **Embase** | **LILACS** | **Tropical Disease Bulletin** |
| --- | --- | --- | --- | --- |
| Date of search | *1st April 2013*  *13^th^ June 2014* | *2^nd^ April 2013*  *13^th^ June 2014* | *3^rd^ April 2013*  *16^th^ June 2014* | *8^th^ April 2013* |
| 1 | Chagas disease (MeSH)  Reduviidae (MeSH) expl  Trypanosomiasis (MeSH)  American trypanosomiasis | Chagas disease (Emt) expl  Reduviidae (Emt) expl  American trypanosomiasis | Chagas disease (DeCS) | ("Chagas disease" or "american trypanosomiasis" or reduviidae) |
| 2 | Dengue (MeSH) expl  Aedes (MeSH) expl | Dengue (Emt) expl  Aedes (Emt) expl | Dengue (DeCS) | (dengue or aedes) |
| 3 | Trypanosomiasis (MeSH)  Trypanosomiasis, African (MeSH) expl  Human African trypanosomiasis  Glossinidae (MeSH) expl | African trypanosomiasis (Emt) expl  Human African trypanosomiasis  Glossinidae (Emt) expl |  | ("human african trypanosomiasis" or "african trypanosomiasis" or "sleeping sickness" or glossinidae) |
| 4 | Encephalitis, Japanese (MeSH) expl  Culex (MeSH) | Japanese encephalitis/ (Emt) expl  Culex (Emt) expl | Encephalitis, Japanese (DeCS) | ("japanese encephalitis" or culex) |
| 5 | Leishmaniasis (MeSH) expl  Kala Azar  Psychodidae (MeSH) expl  Sand fly  Espundia | Leishmaniasis (Emt) expl  Kala azar  Psychodidae (Emt) expl  Sand fly  Espundia | Leishmaniasis (DeCS) | (leishmaniasis or "kala azar" or psychodidae or "sand fly") |
| 6 | Elephantiasis, Filarial (MeSH)  Elephantiasis (MeSH)  Lymphatic filariasis | Lymphatic filariasis (Emt) expl  Lymphatic filariasis | Elephantiasis, filarial (DeCS) | ("lymphatic filariasis" or elephantiasis) |
| 7 | Onchocerciasis (MeSH) expl  River blindness  Roble’s disease  Simuliidae (MeSH)  Black fly | Onchocerciasis (Emt) expl  River blindness  Robles disease  Simuliidae (Emt) expl  Black fly | Onchocerciasis (DeCS) | (onchocerciasis or "river blindness" or simuliidae or "black fly") |
| 8 | Mosquito nets (MeSH) expl  Pyrethrins (MeSH)  Pyreth*  ITN  LLIN  Insecticide treated net  Long lasting insecticide treated net  Bednet  Insecticide-treated  Insecticide-impregnated  Curtain  Hous* improve*  Hous* design  Eaves  House screen*  Ceiling | Bed net (Emt) expl  Pyrethroid (Emt) expl  Insecticide treated net  LLIN  ITN  Insecticide-treated  Insecticide-impregnated  Curtain  Hous* improvement  Hous* design  Eaves  House screen*  Ceiling | Mosquito nets (DECS) Insecticide-Treated Bednets (DECS) | “Vector control” |
