# Supplementary material for: Benefit of Insecticide-Treated Nets, Curtains and Screening on Vector Borne Diseases, Excluding Malaria: A Systematic Review and Meta-analysis
Source: PLoS Negl Trop Dis. 2014 Oct 9;8(10):e3228. doi: 10.1371/journal.pntd.0003228 (PMC4191944; doi:10.1371/journal.pntd.0003228)
Supplement: Supporting Information S2 — Excluded studies and reasons for exclusion. (DOCX) [file pntd.0003228.s002.docx]

**Supporting information S2: Studies excluded from review**

| **Disease** | **Author and date** | **Reason** |
| --- | --- | --- |
| Visceral leishmaniasis | Basimke 1995 [[1](#_ENREF_1)] | Permethrin-treated cloth screens measuring 1 x 9 m were fixed near the bed on the mud walls. Intervention is more akin to insecticide treated wall lining and IRS. |
|  | Courtenay 2007 [[2](#_ENREF_2)]  Dinesh 2008 [[3](#_ENREF_3)]  Mondal 2013 [[4](#_ENREF_4)] | Wrong comparison (ITN vs untreated net) |
|  | Das 2010 [[5](#_ENREF_5)]  Chowdhury 2011 [[6](#_ENREF_6)] | Data from Joshi 2009 paper included since this pools data from all three study sites (only 2 of 3 sites reported separately in Das 2010 and Chowdhury 2011) |
|  | Mondal 2010 [[7](#_ENREF_7)] | Comparison unclear - intervention group had existing nets dipped but unclear what proportion of control group were using un-treated nets. |
|  | Picado 2009 [[8](#_ENREF_8)] | Wrong comparison (Untreated net vs nothing) |
|  | Ritmeijer 2007 [[9](#_ENREF_9)] | Programme with staged roll out of LLINs. No control group. |
| Cutaneous leishmaniasis | Reyburn 2000 [[10](#_ENREF_10)] | Chaddar data excluded – insecticide-treated Islamic cloth used as top sheet |
|  | Jalouk 2007 [[11](#_ENREF_11)]  Tayeh 1997 [[12](#_ENREF_12)] | Wrong comparison (ITN vs untreated net) |
|  | Elnaiem 1999 [[13](#_ENREF_13)] | Non-randomised study and no baseline data reported |
|  | Maroli 1991 [[14](#_ENREF_14)] | Paper reports two field studies with unorthodox designs. In first study CDC light trap is placed under bednet. Second study used cross-over design but no baseline data or control and each catch with treated net compared back to previous night with untreated net. |
|  | Moosa-Kazemi 2007 [[15](#_ENREF_15)] | Wrong comparison (ITN and treated curtain versus nothing) |
|  | Yaghoobi-Ershadi 2006 [[16](#_ENREF_16)] | Wrong comparison (ITN and treated curtain versus nothing / ITN and treated curtain versus untreated net and curtain / Untreated net and curtain versus nothing) |
| Chagas disease | Herber 2003 [[17](#_ENREF_17)] | Wrong comparison (treated vs untreated curtain) |
|  | Wood 1999 [[18](#_ENREF_18)] | No numerical outcomes – reported as infestation / no infestation |
|  | Kroeger 2003 [[19](#_ENREF_19)] | Wrong comparison – treated vs untreated bednets |
|  | Ferral 2010 [[20](#_ENREF_20)] | Non-randomised study and no baseline data reported (looks to be averaged across all sites, not split by intervention and control) |
| Lymphatic filariasis | Ogoma 2010 [[21](#_ENREF_21)] | Entomological data collected in local houses and experimental huts not presented separately |
|  | Ansari 2001 [[22](#_ENREF_22)] | Wrong comparison (treated curtain vs untreated curtain) |
|  | Odermatt 2008 [[23](#_ENREF_23)] | Case control study |
|  | Weerasooriya 1996 [[24](#_ENREF_24)] | Non-randomised study and no baseline data reported |
|  | Pedersen 2002 [[25](#_ENREF_25)] | No control group |
| Dengue | Ansari 2001 [[22](#_ENREF_22)]  Lorono Pino 2013 [[26](#_ENREF_26)]  Madarieta 1999 [[27](#_ENREF_27)] | Wrong comparison (treated curtain vs untreated curtain) |
|  | Vanlerberghe 2011 [[28](#_ENREF_28)] | No control group |
|  | Tsuzuki 2010 [[29](#_ENREF_29)] | Case control study |
| Japanese encephalitis | Dapeng 1994 [[30](#_ENREF_30)] | Non-randomised study and no baseline data reported |
|  | Luo 1994 [[31](#_ENREF_31)] | Case control study |

1. Basimike M, Mutinga MJ (1995) Effects of permethrin-treated screens on phlebotomine sand flies, with reference to Phlebotomus martini (Diptera: Psychodidae). Journal of medical entomology 32: 428-432.

2. Courtenay O, Gillingwater K, Gomes PAF, Garcez LM, Davies CR (2007) Deltamethrin-impregnated bednets reduce human landing rates of sandfly vector Lutzomyia longipalpis in Amazon households. Medical and veterinary entomology 21: 168-176.

3. Dinesh DS, Das P, Picado A, Davies C, Speybroeck N, et al. (2008) Long-lasting insecticidal nets fail at household level to reduce abundance of sandfly vector Phlebotomus argentipes in treated houses in Bihar (India). Tropical Medicine and International Health 13: 953-958.

4. Mondal D, Huda MM, Karmoker MK, Ghosh D, Matlashewski G, et al. (2013) Reducing visceral leishmaniasis by insecticide impregnation of bed-nets, Bangladesh. Emerging Infectious Diseases 19: 1131-1134.

5. Das ML, Roy L, Rijal S, Paudel IS, Picado A, et al. (2010) Comparative study of kala-azar vector control measures in eastern Nepal. Acta tropica 113: 162-166.

6. Chowdhury R, Dotson E, Blackstock AJ, McClintock S, Maheswary NP, et al. (2011) Comparison of insecticide-treated nets and indoor residual spraying to control the vector of visceral leishmaniasis in Mymensingh District, Bangladesh. American Journal of Tropical Medicine and Hygiene 84: 662-667.

7. Mondal D, Chowdhury R, Huda MM, Maheswary NP, Akther S, et al. (2010) Insecticide-treated bed nets in rural Bangladesh: Their potential role in the visceral leishmaniasis elimination programme. Tropical Medicine and International Health 15: 1382-1389.

8. Picado A, Kumar V, Das M, Burniston I, Roy L, et al. (2009) Effect of untreated bed nets on blood-fed Phlebotomus argentipes in kala-azar endemic foci in Nepal and India. Mem Inst Oswaldo Cruz 104: 1183-1186.

9. Ritmeijer K, Davies C, van Zorge R, Wang SJ, Schorscher J, et al. (2007) Evaluation of a mass distribution programme for fine-mesh impregnated bednets against visceral leishmaniasis in eastern Sudan. Tropical Medicine and International Health 12: 404-414.

10. Reyburn H, Ashford R, Mohsen M, Hewitt S, Rowland M (2000) A randomized controlled trial of insecticide-treated bednets and chaddars or top sheets, and residual spraying of interior rooms for the prevention of cutaneous leishmaniasis in Kabul, Afghanistan. Transactions of the Royal Society of Tropical Medicine and Hygiene 94: 361-366.

11. Jalouk L, Al Ahmed M, Gradoni L, Maroli M (2007) Insecticide-treated bednets to prevent anthroponotic cutaneous leishmaniasis in Aleppo Governorate, Syria: results from two trials. Transactions of the Royal Society of Tropical Medicine and Hygiene 101: 360-367.

12. Tayeh A, Jalouk L, Al-Khiami AM (1997) A cutaneous leishmaniasis control trial using pyrethroid-impregnated bednets in villages near Aleppo, Syria. World Health Organization.

13. Elnaiem DA, Aboud MA, El Mubarek SG, Hassan HK, Ward RD (1999) Impact of pyrethroid-impregnated curtains on Phlebotomus papatasi sandflies indoors at Khartoum, Sudan. Medical and veterinary entomology 13: 191-197.

14. Maroli M, Majori G (1991) Permethrin-impregnated curtains against phlebotomine sandflies (Diptera: Psychodidae): laboratory and field studies. Parassitologia 33 Suppl: 399-404.

15. Moosa-Kazemi SH, Yaghoobi-Ershadi MR, Akhavan AA, Abdoli H, Zahrael-Ramazani AR, et al. (2007) Deltamethrin-impregnated bed nets and curtains in an anthroponotic cutaneous leishmaniasis control program in northeastern Iran. Annals of Saudi Medicine 27: 6-12.

16. Yaghoobi-Ershadi MR, Moosa-Kazemi SH, Zahraei-Ramazani AR, Jalai-Zand AR, Akhavan AA, et al. (2006) Evaluation of deltamethrin-impregnated bed nets and curtains for control of zoonotic cutaneous leishmaniasis in a hyperendemic area of Iran. [French]. Bulletin de la Societe de pathologie exotique 99: 43-48.

17. Herber O, Kroeger A (2003) Pyrethroid-impregnated curtains for Chagas' disease control in Venezuela. Acta tropica 88: 33-38.

18. Wood E, De Licastro SA, Casabe N, Picollo MI, Alzogaray R, et al. (1999) A new tactic for Triatoma infestans control: Fabrics impregnated with beta-cypermethrin. Revista Panamericana de Salud Publica/Pan American Journal of Public Health 6: 1-7.

19. Kroeger A, Villegas E, Ordonez-Gonzalez J, Pabon E, Scorza JV (2003) Prevention of the transmission of Chagas' disease with pyrethroid-impregnated materials. The American journal of tropical medicine and hygiene 68: 307-311.

20. Ferral J, Chavez-Nunez L, Euan-Garcia M, Ramirez-Sierra MJ, Najera-Vazquez MR, et al. (2010) Comparative field trial of alternative vector control strategies for non-domiciliated Triatoma dimidiata. American Journal of Tropical Medicine and Hygiene 82: 60-66.

21. Ogoma SB, Lweitoijera DW, Ngonyani H, Furer B, Russell TL, et al. (2010) Screening mosquito house entry points as a potential method for integrated control of endophagic filariasis, arbovirus and malaria vectors. PLoS Neglected Tropical Diseases 4.

22. Ansari MA, Razdan RK (2001) Concurrent control of mosquitoes and domestic pests by use of deltamethrin-treated curtains in the New Delhi Municipal Committee, India. Journal of the American Mosquito Control Association 17: 131-136.

23. Odermatt P, Leang R, Bin B, Bunkea T, Socheat D (2008) Prevention of lymphatic filariasis with insecticide-treated bednets in Cambodia. Annals of tropical medicine and parasitology 102: 135-142.

24. Weerasooriya MV, Munasinghe CS, Mudalige MPS, Curtis CF, Samarawickrema WA (1996) Comparative efficacy of house curtains impregnated with permethrin, lambdacyhalothrin or bendiocarb against the vector of bancroftian filariasis, Culex quinquefasciatus, in Matara, Sri Lanka. Transactions of the Royal Society of Tropical Medicine and Hygiene 90: 103-104.

25. Pedersen EM, Mukoko DA (2002) Impact of insecticide-treated materials on filaria transmission by the various species of vector mosquito in Africa. Annals of tropical medicine and parasitology 96 Suppl 2: S91-95.

26. Lorono-Pino MA, Garcia-Rejon JE, Machain-Williams C, Gomez-Carro S, Nunez-Ayala G, et al. (2013) Towards a Casa Segura: A Consumer Product Study of the Effect of Insecticide-Treated Curtains on Aedes aegypti and Dengue Virus Infections in the Home. Am J Trop Med Hyg.

27. Madarieta SK, Salarda A, Benabaye MRS, Bacus MB, Tagle JR (1999) Use of permethrin-treated curtains for control of Aedes aegypti in the Philippines. . Dengue Bull 23: 51-54.

28. Vanlerberghe V, Villegas E, Oviedo M, Baly A, Lenhart A, et al. (2011) Evaluation of the effectiveness of insecticide treated materials for household level dengue vector control. PLoS Neglected Tropical Diseases 5: e994.

29. Tsuzuki A, Thiem VD, Suzuki M, Yanai H, Matsubayashi T, et al. (2010) Can daytime use of bed nets not treated with insecticide reduce the risk of dengue hemorrhagic fever among children in Vietnam? The American journal of tropical medicine and hygiene 82: 1157-1159.

30. Dapeng L, Renguo Y, Jinduo S, Hongru H, Ze W (1994) The effect of DDT spraying and bed nets impregnated with pyrethroid insecticide on the incidence of Japanese encephalitis virus infection. Transactions of the Royal Society of Tropical Medicine and Hygiene 88: 629-631.

31. Luo D, Zhang K, Song J, Yao R, Huo H, et al. (1994) The protective effect of bed nets impregnated with pyrethroid insecticide and vaccination against Japanese encephalitis. Transactions of the Royal Society of Tropical Medicine & Hygiene 88: 632-634.
