# Supplementary material for: Benefit of Insecticide-Treated Nets, Curtains and Screening on Vector Borne Diseases, Excluding Malaria: A Systematic Review and Meta-analysis
Source: PLoS Negl Trop Dis. 2014 Oct 9;8(10):e3228. doi: 10.1371/journal.pntd.0003228 (PMC4191944; doi:10.1371/journal.pntd.0003228)
Supplement: Supporting Information S3 — Data extraction form. (DOCX) [file pntd.0003228.s003.docx]

**Supporting information S3: Data Extraction Form**

| **S.No.** | **Variable Name** | **Variable Description** |
| --- | --- | --- |
| ***Publication Information*** | | |
| 1 | Reference ID | Unique identifier assigned for a publication |
| 2 | Author | Authors of publication |
| 3 | Publication year | Year of publication |
| 4 | Journal | Journal/Medium in which results are published |
| 5 | Volume | Volume of the Journal |
| 6 | Issue | Issue number of the Journal |
| 7 | Pages | Start page and end page of Journal |
| 8 | Title | Title of publication or source |
| 9 | Trial number | Unique identifier for the trial. |
| ***Trial Information*** | | |
| 10 | Trial start year | Calendar year in which trial was started (for example, baseline measurements) |
| 11 | Trial end year | Year of trial completion |
| 12 | Countries | Geographical location where the study is conducted |
| 13 | Number of arms | Number of treatment arms |
| 14 | Controlled trial | Does the trial have any control arm or not? |
| 15 | Randomisation | Is the trial randomised? Yes/no/uncertain |
| 16 | Level at which intervention was allocated | Individual / house / village / zone |
| 17 | Allocation | How were interventions allocated? |
| 18 | Blinding | Please state level of blinding - investigator, recipient of intervention, outcome assessor |
| 19 | Trial design | Design adopted for the trial: i) randomised controlled trial, ii) before-after trial (non-randomised)  iii) rotational design e.g. 4 houses and types of net, each house receives each net for 1 night, similarly for personal repellents, iv) other…. |
| 20 | Trial Comments | Overall trial comments if any |
| 21 | Analysis took account of cluster randomised design | Analysis section specifically mentions that they took account of clustering. NB: only relevant if study is cluster randomised |
| ***Background Information*** | | |
| 22 | Disease | Which disease are the authors trying to control using the intervention? |
| 23 | Incidence rate in location | Incidence rate of disease condition in mentioned location (baseline or historic) |
| 24 | Transmission season | When is the main disease transmission season (if reported) |
| 25 | Region | Exact location where study was conducted (name of town/s or villages and province, longitude and latitude if given) |
| 26 | Urban/Rural | Area of the trial conducted |
| 27 | Vector Species 1 | Name of the main vector species |
| 28 | Vector Species 2 | Name of the main vector species |
| 29 | Vector Species 3 | Name of the main vector species |
| ***Treatment Description*** | | |
| 32 | Treatment Arm 1 | Type of treatment (NB: There may be multiple types of treatment within arms). E.g. treated bednet, indoor residual spraying etc |
| 33 | Treatment 1 Description | Additional information available regarding the treatment (chemical / concentration / net mesh size / residual activity of insecticide etc) |
| 34 | Treatment 1: Number of clusters? | Number of clusters (individual / house / village / zone) that received intervention |
| 35 | Treatment 1: Name of clusters / area | Name of clusters / area that received treatment |
| 36 | Treatment 1: Number of times treatment administered |  |
| 37 | Treatment 1: Date treatment was administered |  |
| REPEAT IF MORE THAN ONE TREATMENT ARM | | |
| 38 | Control arm | Name of the control |
| 39 | Control description | Additional information available regarding the control |
| 40 | Number of clusters? | Number of clusters (individual / house / village / zone) that received control |
| 41 | Name of control clusters / area | Name of clusters / area that received control |
| 42 | Number of times control administered |  |
| 43 | Date control was administered |  |
| ***Outcome Measurement - entomological*** | | |
| 44 | Outcome measures assessed in study | Which outcome measures were assessed in the study? Entomological / clinical / both |
| 45 | Entomological outcome | E.g. entomological inoculation rate, biting/landing density or mosquito abundance (no./trap or house). |
| 46 | Length of baseline period | State total length and dates |
| 47 | Length of post-intervention period | State total length and dates |
| 48 | Post intervention period duration | Does the post intervention period cover a whole year or transmission season? |
| 49 | Sampling method | Method used for sampling (e.g. human landing catch, aspirator, sticky trap, CDC light trap) |
| 50 | Detail on sampling method | Description of sampling method |
| 51 | Number of traps / houses | Number of traps total and number of traps per each sampling site |
| 52 | How were the sampling sites chosen? | Describe how the sites were chosen for sampling. Does the paper say they were chosen randomly? How was the random selection performed? Is this described? Or was the sampling done in ALL houses? |
| 53 | Number of times outcome was measured during baseline period |  |
| 54 | Number of times outcome was measured during post-intervention period |  |
| 55 | Outcome Location | Location of the outcome. Eg: Figure 2, Table 1 etc. |
| REPEAT IF MORE THAN ONE ENTOMOLOGICAL OUTCOME ASSESSED | | |
| ***Outcome Measurement - clinical*** | | |
| 89 | Clinical outcome | Clinical outcome assessed (e.g. prevalence of infection) |
| 90 | Patient characteristics | For example; age, previous infection (immunity) etc |
| 91 | Length of baseline period |  |
| 92 | Number of times outcome was measured during baseline period |  |
| 93 | Length of post-intervention period |  |
| 94 | Number of times outcome was measured during post-intervention period |  |
| 95 | Post intervention period duration |  |
| 96 | Method of measuring clinical outcome | Clinical judgement / diagnostic test used |
| 97 | Outcome Location | Location of the outcome. Eg: Figure 2, Table 1 etc. |
| REPEAT IF MORE THAN ONE CLINICAL OUTCOME ASSESSED | | |
| Tables and figures from published paper: | | |
