# Supplementary material for: Benefit of Insecticide-Treated Nets, Curtains and Screening on Vector Borne Diseases, Excluding Malaria: A Systematic Review and Meta-analysis
Source: PLoS Negl Trop Dis. 2014 Oct 9;8(10):e3228. doi: 10.1371/journal.pntd.0003228 (PMC4191944; doi:10.1371/journal.pntd.0003228)
Supplement: Supporting Information S5 — Risk of bias assessment form. (DOCX) [file pntd.0003228.s005.docx]

**Supporting information S5: Risk of Bias Assessment Form**

| **Criterion** | **Type of bias** | **Explanation** | | |
| --- | --- | --- | --- | --- |
|  |  | **Low risk of bias** | **High risk of bias** | **Unclear** |
| **GENERAL CONSIDERATIONS** | | | | |
| Sequence generation | Selection bias | Randomised study and random nature of sequence generation well described OR rotational design study where each individual / house has received every intervention at least once . | Study is non-randomised OR non-random method of sequence generation used OR rotational design study where each individual / house has not received every intervention at least once. | No or unclear information reported (e.g. paper states study is randomised but method of sequence generation is not described). |
| Allocation concealment | Selection bias | Patients and investigators could not foresee assignment. | Inadequate concealment of allocations prior to assignment. | No or unclear information reported. |
| Blinding (performance) | Performance bias | Participants and personnel were not aware of which intervention they were allocated to during the study. | Performance bias due to knowledge of the allocated interventions by participants and personnel during the study. | No or unclear information reported. |
| Contamination | - | It is unlikely that the control group received the intervention. | Control group may have inadvertently received the intervention (e.g. proximity of control and intervention areas or insufficient washout period during crossover or rotational design study). | No or unclear information reported. |
| Selective outcome reporting | Reporting bias | Outcomes of interest clearly stated and all pre-specified outcomes are reported. | Not all pre-specified outcomes are reported, or additional outcomes are reported. | Unclear or NA (Outcomes not pre-specified in a published protocol). |
| Incorrect analysis | - | Correct analysis technique utilised (e.g. clustering taken into account in analysis for cluster-randomised trials or appropriate technique used for repeated measures). | Incorrect analysis technique utilised.  (e.g. clustering not taken into account in analysis for cluster-randomised trials or inappropriate technique used for repeated measures). | No or unclear information reported. |
| **CLINICAL OUTCOMES** | | | | |
| Baseline characteristics | Selection bias | Baseline characteristics reported to be similar in control and intervention areas. | Significant differences in baseline characteristics between control and intervention areas. | No or unclear information reported. |
| Blinding (Detection) | Detection bias | Outcome assessors blinded to intervention allocation. | Detection bias due to knowledge of the allocated interventions by outcome assessors. | No or unclear information reported. |
| Incomplete outcome data | Attrition bias | No or low missing data (<20%), reason for missing data is unlikely to be related to the true outcome, or missing data is balanced across groups. | High missing data (>20%), missing data is likely to be related to the true outcome, or missing data is unbalanced across groups. | No or unclear information reported. |
| Recruitment bias | Recruitment bias | No change in size or number of clusters after randomisation | Possible change in size or number of clusters after randomisation | No or unclear information reported |
| Other biases (confounding) | - | Non randomised studies: no evidence of confounding (selection bias) | Non randomised studies: evidence of confounding (selection bias) |  |
| **ENTOMOLOGICAL OUTCOMES** | | | | |
| Baseline characteristics | Selection bias | Pre-post design or randomised controlled trial:  Baseline entomological data available for previous transmission season (seasonal transmission) or at least 3-6 months (year round transmission) | Pre-post design or randomised controlled trial:  No baseline entomological data OR baseline entomological data available for short time period only (less than one transmission season – seasonal transmission or <3-6 months – year round transmission). | No or unclear information reported |
|  |  | Rotational design:  Each individual / house receives every intervention at least once. | Rotational design:  Each individual / house has not received every intervention at least once. |  |
| Blinding (Detection) | Detection bias | Investigators / data collectors blinded to intervention allocation OR  Objective measurement technique (e.g. CDC light trap, odour-baited trap, sticky trap) utilised for data collection | Non-standardised measurement technique (human landing catches, aspirator & knock down catches) utilised for data collection.  OR Efficiency of sampling technique likely to vary between study arms (e.g. CDC light trap collection – bednet versus no bednet) | No or unclear information reported |
| Random selection of sites for entomological monitoring | Detection bias | Units for entomological sampling selected randomly and random nature of sequence generation well described OR entomological sampling done in all units | Units for entomological sampling not selected randomly | No or unclear information reported |
