# Supplementary material for: Benefit of Insecticide-Treated Nets, Curtains and Screening on Vector Borne Diseases, Excluding Malaria: A Systematic Review and Meta-analysis
Source: PLoS Negl Trop Dis. 2014 Oct 9;8(10):e3228. doi: 10.1371/journal.pntd.0003228 (PMC4191944; doi:10.1371/journal.pntd.0003228)
Supplement: Supporting Information S6 — Study quality assessment form. (DOCX) [file pntd.0003228.s006.docx]

**Supporting information S6: Study Quality Assessment Form**

| Study design |  | Lower if: | Quality score |
| --- | --- | --- | --- |
| Randomised controlled trial (score +10) |  | Sample size calculation:  0 sample size calculation performed or significant effect of intervention shown (beneficial or otherwise)  -1 sample size calculation not performed and no significant effect of intervention shown (beneficial or otherwise) | ≥ 7 high quality  ≥4 < 7 medium quality  <4 low quality |
| Cross over or rotational study (score +7) |  | Sample size calculation (entomological outcomes):  0 sample size calculation performed  -0.5 not performed  -1 < 10 sampling sites per arm |  |
| Pre-post study (score +4) |  | Length of follow up period for entomological outcomes:  0 > 1 year or transmission season  -0.5 > 1 year or transmission season but limited repeat measures during this time  -1 < 1 year or transmission season |  |
|  |  | Risk of bias  -0.5 medium  -1 high |  |
