# Supplementary material for: Benefit of Insecticide-Treated Nets, Curtains and Screening on Vector Borne Diseases, Excluding Malaria: A Systematic Review and Meta-analysis
Source: PLoS Negl Trop Dis. 2014 Oct 9;8(10):e3228. doi: 10.1371/journal.pntd.0003228 (PMC4191944; doi:10.1371/journal.pntd.0003228)
Supplement: Supporting Information S7 — Characteristics of studies identified. (DOCX) [file pntd.0003228.s007.docx]

**Supporting information S7: Characteristics of Studies Identified**

**Characteristics of cutaneous leishmaniasis studies**

| Author and date | Study Type | No. of subjects / areas | Country | Intervention | Insecticide resistance | 1° outcome | 2°outcome |
| --- | --- | --- | --- | --- | --- | --- | --- |
| Alexander 1995 [[1](#_ENREF_1)] | Rotational design | 3 study houses – treatments and collectors rotated | Colombia | Deltamethrin impregnated bednets (26 mg a.i./m^2^. and mesh size 64 per cm^2^). | Bioassay: 1 min  exposure = 35% knock down within 1h / 66% mortality within 24h. 2 min exposure = 69% knock down within 1h / 100% mortality within 24h. | Sandfly density | - |
|  |  |  |  | Deltamethrin impregnated curtains (26 mg a.i./m^2^. and mesh size 64 per cm^2^). Impregnated curtains were suspended across the doorways and windows of rooms. |  |  |  |
| Alten 2003 [[2](#_ENREF_2)] | Non-randomised controlled pre-post study | 4 clusters (2 control, 2 ITN).  Population =  3761 ITN and 5636 Control | Turkey | Deltamethrin impregnated bednets (25 mg a.i./m^2^ and mesh size 156 per cm^2^, 100 denier). Re-impregnated every 6 months by the field team. | Not reported / assessed. | Incidence of cutaneous leishmaniasis (questionnaire only, no mention of diagnostic test / clinical examination) | Sandfly density |
| Emami 2009 [[3](#_ENREF_3)] | Cluster-RCT | 12 sectors across 2 cities. Total population = 8,620  Total households = 3000 | Iran | Olyset® LLIN (polyethylene net impregnated with 2% permethrin) | Not reported / assessed. | Incidence of cutaneous leishmaniasis (questionnaire, clinical examination, hospital record check) | Sandfly density |
| Kroeger 2002 [[4](#_ENREF_4)] | Cluster-RCT | 14 sectors (7 intervention / 7 control). Between 53 and 509 participants per sector. Total participants =  2913 from 569 houses | Venezuela | Lambdacyhalothrin impregnated curtains (12.5 mg/m^2^, mesh size = 0.05 mm. Curtains were fitted to the windows of houses and were re-impregnated after six months. | Not reported / assessed. | Incidence of cutaneous leishmaniasis  (questionnaire, clinical examination, hospital record check) | Sandfly density |
| Majori 1989 [[5](#_ENREF_5)] | Controlled, pre-post crossover study (analysed as if pre-post study) | 2 houses – impregnated curtains rotated between houses (3 total, also non impregnated curtains) | Burkina Faso | Permethrin impregnated curtains  (1g/m^2^, 0.5cm mesh). Curtains installed inside doorways and under the eaves. | Not reported / assessed. | Sandfly density | - |
| Nadim 1995 [[6](#_ENREF_6)] | Cluster-RCT | 2 clusters/ areas  Total population = 2,414 | Iran | Deltamethrin impregnated bednets (25mg per m^2^) | Not reported / assessed for vector species (bioassay using *Anopheles stephensi* only). | Incidence of cutaneous leishmaniasis (questionnaire and clinical examination) | Sandfly density (data not extractable) |
| Noazin 2013 [[7](#_ENREF_7)] | Non-randomised controlled pre-post study | 2 clusters/ areas  Intervention = 6546 houses (~23,000 pop.) Control = 22,355 houses (61,224 pop.) | Iran | Deltamethrin impregnated screens and curtains (25mg per m^2^, polyester fabric 156 holes/in^2^). Covered all windows with treated screens and entrances to the interior of the house with treated curtains. | Not reported / assessed. | Incidence of cutaneous leishmaniasis (Microscopic examination of scraping from skin lesion) | - |
| Reyburn 2000 [[8](#_ENREF_8)] | Cluster-RCT | Clusters of 10 households each  Population =  1195 ITN and 1759 control | Afghanistan | Permethrin impregnated bednets (0.5 g/m^2^, 100-denier, 156 holes/mesh per in^2^) | Not reported / assessed. | Incidence of cutaneous leishmaniasis  (clinical examination) | - |
| Rojas 2006 [[9](#_ENREF_9)] | Cluster-RCT | 20 clusters (villages)  Population =  1791 ITN and 1,840 control | Colombia | Deltamethrin impregnated bednets (K-Othrine E-25® (deltamethrin and 35 holes per cm^2^) and two bars of repellents (20 % DEET + 5% permethrin) and tree trunks painted with white wash and educational program. Bednets re-impregnated every 3 months.  N.B. Use of repellent (by last survey only 5% of participants were using it every day) and white washing of tree trunks declined rapidly so can attribute effects mainly to bednets. | Not reported / assessed. | Incidence of cutaneous leishmaniasis  (clinical examination and leishmanin skin test) | - |

**Characteristics of visceral leishmaniasis studies**

| Author and date | Study Type | No. of subjects / areas | Country | Intervention | Insecticide resistance | 1° outcome | 2°outcome |
| --- | --- | --- | --- | --- | --- | --- | --- |
| Elnaiem 1999 [[10](#_ENREF_10)] | Rotational design | Single site – collectors rotated round | Sudan | Lambda-cyhalothrin (ICON®) impregnated bednets (10mg a.i./m^2^, 100 denier, 156 mesh), hand treated | Bioassay: 30 sec exposure = 100% mortality of *P. orientalis* within 1h | Sandfly (*P. orientalis*) density | - |
| Joshi 2009 [[11](#_ENREF_11)] | Cluster-RCT | In each of the 4 study sites: Bangladesh (one), India (one) and Nepal (two) six clusters were randomly assigned to each of the study interventions.  Each cluster = 50 – 100 houses | Bangladesh, India and Nepal | PermaNet® LLIN (Vestergaard-Frandsen Company, Lausanne, Switzerland) with small mesh (156 holes/in^2^), polyester, resin coating containing deltamethrin (55 mg/m^2^) | Bioassay of wild caught *P. argentipes*: 3 min exposure = > 80% mortality within 24h in all sites | Sandfly density | - |
| Picado 2010 [[12](#_ENREF_12),[13](#_ENREF_13)] | Cluster-RCT | 26 clusters  Population =  10,563 LLIN and 10,704 control | India and Nepal | PermaNet® 2.0 LLIN (75 denier, 25 holes/cm^2^, with deltamethrin (55 mg/m^2^) coated fibres) | Bioassay of wild caught *P. argentipes:*60 min exposure = >95% mortality within 24h in both sites [[14](#_ENREF_14)] | Number of incident *L. donovani* infections (seroconversion using direct agglutination test) | Number of incident cases of visceral leishmaniasis (clinical examination and rapid diagnostic test)  Density of sandflies (*P. argentipes*) |

**Characteristics of lymphatic filariasis studies**

| Author and date | Study Type | No. of subjects / areas | Country | Intervention | Insecticide resistance | 1° outcome | 2°outcome |
| --- | --- | --- | --- | --- | --- | --- | --- |
| Bøgh 1998 [[15](#_ENREF_15)] | Cluster-RCT | 12 villages – populations ranged from 375 to 1151 inhabitants. | Kenya | Permethrin impregnated bednets (100 denier, mesh 156, 500 mg/m^2^ permethrin). Nets were re-impregnated every six months. | Not reported / assessed. | Mosquito density (indoor and outdoor resting, split by species) | - |
| Charlwood 1987 [[16](#_ENREF_16)] | Rotational design | 3 study houses - treatments and collectors rotated | Papua New Guinea | Permethrin impregnated bednets (0.5g/m^2^) | Not reported / assessed. | Mosquito density (indoor resting) | - |
| Poopathi 1995 [[17](#_ENREF_17)] | Non-randomised controlled pre- post study | 12 huts with high indoor resting density of mosquitoes selected:  8 with impregnated curtains, 2 with untreated curtains, 2 with nothing. | India | Deltamethrin impregnated curtains (50 mg/m^2^, hessian fabric, mesh size of 5.5 x 5.5 mm). Curtains hung in the eaves and doorways. | Bioassay: 3 min exposure to freshly treated curtains = 100% mortality of  both lab reared *An.stephensi* and *Cx. quinquefasciatus*  females after 24h. 3 min exposure 10-16 wks post treatment = <50% mortality after 24h | Mosquito density (indoor resting and man biting) | - |

**Characteristics of dengue studies**

| Author and date | Study Type | No. of subjects / areas | Country | Intervention | Insecticide resistance | 1° outcome | 2°outcome |
| --- | --- | --- | --- | --- | --- | --- | --- |
| Kroeger 2006 [[18](#_ENREF_18)] | Cluster- RCT | Total participants = 4743 inhabitants (1095 houses) in Veracruz | Mexico (Veracruz site) | Lambdacyhalothrin impregnated curtains (15 mg/m^2^ netting, treated by hand). Curtains were hung loosely at the windows (mean 2.8 curtains per household). N.B. Intervention also included water treatment with pyriproxyfen chips. However, people didn’t accept or use the pyriproxyfen chips, so changes can be attributed to curtains mainly. | Not reported / assessed. | Breteau index, house index, pupae per person | - |
| Lenhart 2008 [[19](#_ENREF_19)] | Cluster-RCT | Total study area = 1017 houses, divided into 18 sectors (clusters) with approximately equal number of houses in each sector. | Haiti | Olyset® LLIN (2% permethrin) | Bioassay using lab reared *A. aegypti:* 2 min exposure to new bednets = 50% knock down after 60 min, 30% mortality after 24h. 2 min exposure to 5 mth (or 10mth) old bednets = 80% (37%) knock down after 60 mins, 34% (15%) mortality after 24h. | House index, container index, Breteau index, pupae per person, ovitrap positivity | IgM serostatus (but no control data so not extractable) |
| Lenhart 2013 [[20](#_ENREF_20)] | Cluster RCT | Total of 2,037 houses in 26 clusters | Thailand | Deltamethrin impregnated window curtains (55 mg/m^2^, made from PermaNet® polyester LLIN netting, factory treated with long-lasting deltamethrin formulation). Sufficient number of curtains were provided to each house to hang in every window, regardless of the presence or absence of other window coverings. | WHO standard bioassay using mosquitoes reared from eggs collected in oviposition traps: 6 mth timepoint = 84% mortality in ITC and 90% in control clusters, 9 mth timepoint = 92% mortality in ITC and 92% in control clusters. | House index, container index, Breteau index, pupae per person, oviposition rates | - |
| Nguyen 1996 / Igarashi 1997 [[21](#_ENREF_21),[22](#_ENREF_22)] | Non-randomised controlled pre- post study | 2 villages – intervention village had 500 houses (unclear how many in control village) | Vietnam | Olyset® net (LLIN) installed as screens fitting windows, entrances and ventilation openings near the ceilings of the houses in the experimental area. | 100% knock down after 9-12 min exposure to new nets. This effect remained 100% after 8 months. | House index (HI), density index (adults) | IgM serostatus (IgM-capture ELISA) |
| Vanlerberghe 2013 [[23](#_ENREF_23)] | Cluster RCT | 22 clusters – intervention (total of 2032 houses)  66 clusters – control (each 10 houses, total approx. 660 houses) | Thailand | Deltamethrin impregnated window and door curtains (55 mg/m^2^, made from PermaNet® polyester LLIN netting, factory treated with long-lasting deltamethrin formulation). | WHO tube bioassay on *Aedes* sp. (eggs reared to adults in the insectary): 87% mortality pre- and 84% post-intervention. | House index (HI), Breteau index (BI), pupae per person index | - |

**Characteristics of Japanese encephalitis studies**

| Author and date | Study Type | No. of subjects / areas | Country | Intervention | Insecticide resistance | 1° outcome | 2°outcome |
| --- | --- | --- | --- | --- | --- | --- | --- |
| Dutta 2011 [[24](#_ENREF_24)] | Non-randomised controlled pre- post study | 2 localities (clusters) each consisting of 2-3 villages: intervention = 560 ITNs distributed, population / no. of houses unclear. | India | Deltamethrin impregnated bednets  [25 mg/m^2^, Deltamethrin 2.5% (K-othrine®, Bayer Cropscience India Limited, Gujrat, India) emulsifiable concentrate (EC), 156 holes/in^2^] | Not reported / assessed. | Mosquito density (by species). Catches performed in cattle sheds, mosquitoes resting in or around sheds or landing on animals) | IgM serostatus (IgM- capture ELISA) |

**References**

1. Alexander B, Usma MC, Cadena H, Quesada BL, Solarte Y, et al. (1995) Evaluation of deltamethrin-impregnated bednets and curtains against phlebotomine sandflies in Valle del Cauca, Colombia. Medical and Veterinary Entomology 9: 279-283.

2. Alten B, Caglar SS, Kaynas S, Simsek FM (2003) Evaluation of protective efficacy of K-OTAB impregnated bednets for cutaneous leishmaniasis control in Southeast Anatolia-Turkey. Journal of Vector Ecology 28: 53-64.

3. Emami MM, Yazdi M, Guillet P (2009) Efficacy of Olyset long-lasting bednets to control transmission of cutaneous leishmaniasis in Iran. Eastern Mediterranean Health Journal 15: 1075-1083.

4. Kroeger A, Avila EV, Morison L (2002) Insecticide impregnated curtains to control domestic transmission of cutaneous leishmaniasis in Venezuela: Cluster randomised trial. British Medical Journal 325: 810-813.

5. Majori G, Maroli M, Sabatinelli G, Fausto AM (1989) Efficacy of permethrin-impregnated curtains against endophilic phlebotomine sandflies in Burkina Faso. Medical and Veterinary Entomology 3: 441-444.

6. Nadim A, Motabar M, Houshmand B, Keyghobadi K, Aflatonian MR (1995) Evaluation of pyrethroid impregnated bednets for control of anthroponotic cutaneous leishmaniasis in Bam (Islamic Republic of Iran). Geneva: World Health Organization.

7. Noazin S, Shirzadi MR, Kermanizadeh A, Yaghoobi-Ershadi MR, Sharifi I (2013) Effect of large-scale installation of deltamethrin-impregnated screens and curtains in Bam, a major focus of anthroponotic cutaneous leishmaniasis in Iran. Transactions of the Royal Society of Tropical Medicine and Hygiene 107: 444-450.

8. Reyburn H, Ashford R, Mohsen M, Hewitt S, Rowland M (2000) A randomized controlled trial of insecticide-treated bednets and chaddars or top sheets, and residual spraying of interior rooms for the prevention of cutaneous leishmaniasis in Kabul, Afghanistan. Transactions of the Royal Society of Tropical Medicine and Hygiene 94: 361-366.

9. Rojas CA, Weigle KA, Tovar R, Morales AL, Alexander B (2006) A multifaceted intervention to prevent American cutaneous leishmaniasis in Colombia: results of a group-randomized trial. Biomedica 26 Suppl 1: 152-166.

10. Elnaiem DA, Elnahas AM, Aboud MA (1999) Protective efficacy of lambdacyhalothrin-impregnated bednets against *Phlebotomus orientalis*, the vector of visceral leishmaniasis in Sudan. Medical and Veterinary Entomology 13: 310-314.

11. Joshi AB, Das ML, Akhter S, Chowdhury R, Mondal D, et al. (2009) Chemical and environmental vector control as a contribution to the elimination of visceral leishmaniasis on the Indian subcontinent: Cluster randomized controlled trials in Bangladesh, India and Nepal. BMC Medicine 7: 54.

12. Picado A, Das ML, Kumar V, Kesari S, Dinesh DS, et al. (2010) Effect of village-wide use of long-lasting insecticidal nets on visceral Leishmaniasis vectors in India and Nepal: A cluster randomized trial. PLoS Neglected Tropical Diseases 4: e587.

13. Picado A, Singh SP, Rijal S, Sundar S, Ostyn B, et al. (2010) Longlasting insecticidal nets for prevention of *Leishmania donovani* infection in India and Nepal: paired cluster randomised trial. British Medical Journal (Clinical research ed) 341: c6760.

14. Dinesh DS, Das ML, Picado A, Roy L, Rijal S, et al. (2010) Insecticide Susceptibility of *Phlebotomus argentipes* in Visceral Leishmaniasis Endemic Districts in India and Nepal. PLoS Neglected Tropical Diseases 4: e859.

15. Bøgh C, Pedersen EM, Mukoko DA, Ouma JH (1998) Permethrin-impregnated bednet effects on resting and feeding behaviour of lymphatic filariasis vector mosquitoes in Kenya. Medical and Veterinary Entomology 12: 52-59.

16. Charlwood JD, Dagoro H (1987) Impregnated bed nets for the control of filariasis transmitted by *Anopheles punctulatus* in rural Papua New Guinea. Papua and New Guinea Medical Journal 30: 199-202.

17. Poopathi S, Rao DR (1995) Pyrethroid-impregnated hessian curtains for protection against mosquitoes indoors in south India. Medical and Veterinary Entomology 9: 169-175.

18. Kroeger A, Lenhart A, Ochoa M, Villegas E, Levy M, et al. (2006) Effective control of dengue vectors with curtains and water container covers treated with insecticide in Mexico and Venezuela: Cluster randomised trials. British Medical Journal 332: 1247-1250.

19. Lenhart A, Orelus N, Maskill R, Alexander N, Streit T, et al. (2008) Insecticide-treated bednets to control dengue vectors: Preliminary evidence from a controlled trial in Haiti. Tropical Medicine and International Health 13: 56-67.

20. Lenhart A, Trongtokit Y, Alexander N, Apiwathnasorn C, Satimai W, et al. (2013) A cluster-randomized trial of insecticide-treated curtains for dengue vector control in Thailand. American Journal of Tropical Medicine and Hygiene 88: 254-259.

21. Igarashi A (1997) Impact of dengue virus infection and its control. FEMS Immunology and Medical Microbiology 18: 291-300.

22. Nguyen HT, Tien TV, Tien NC, Ninh TU, Hoa NT (1996) The effect of Olyset net screen to control the vector of dengue fever in Viet Nam. Dengue Bulletin 20: 87-92.

23. Vanlerberghe V, Trongtokit Y, Jirarojwatana S, Jirarojwatana R, Lenhart A, et al. (2013) Coverage-dependent effect of insecticide-treated curtains for dengue control in Thailand. American Journal of Tropical Medicine and Hygiene 89: 93-98.

24. Dutta P, Khan SA, Khan AM, Borah J, Sarmah CK, et al. (2011) The effect of insecticide-treated mosquito nets (ITMNs) on Japanese encephalitis virus seroconversion in pigs and humans. American Journal of Tropical Medicine and Hygiene 84: 466-472.
