# Supplementary material for: Benefit of Insecticide-Treated Nets, Curtains and Screening on Vector Borne Diseases, Excluding Malaria: A Systematic Review and Meta-analysis
Source: PLoS Negl Trop Dis. 2014 Oct 9;8(10):e3228. doi: 10.1371/journal.pntd.0003228 (PMC4191944; doi:10.1371/journal.pntd.0003228)
Supplement: Supporting Information S8 — Assessment of risk of bias. (DOCX) [file pntd.0003228.s008.docx]

**Supporting information S8: Assessment of risk of bias**

| Study | General considerations | | | | | | Clinical outcomes | | | | | Entomological outcomes | | | **Overall rating** |
| --- | --- | --- | --- | --- | --- | --- | --- | --- | --- | --- | --- | --- | --- | --- | --- |
|  | Sequence generation | Allocation concealment | Blinding (performance) | Contamination | Outcome reporting | Incorrect analysis | Baseline characteristics | Blinding (Detection) | Incomplete outcome data | Recruitment bias | Other biases (confounding) | Baseline characteristics | Blinding (Detection) | Monitoring site selection |  |
| **Cutaneous leishmaniasis** | | | | | | | | | | | | | | | |
| Alexander 1995 | L | NA | H | L | NA | L | - | - | - | - | - | L | H | L | **L** |
| Alten 2003 | U | U | H | L | NA | H | U | H | U | L | NA | H | L | U | **H** |
| Emami 2009 | L | U | H | L | NA | L | H | H | U | L | NA | H | L | U | **L** |
| Kroeger 2002 | L | U | H | L | NA | L | L | H | L | L | NA | L | L | L | **L** |
| Majori 1989 | U | U | H | NA | NA | L | - | - | - | - | - | H | H | L | **H** |
| Nadim 1995 | L | U | H | L | NA | L | L | U | L | L | NA | - | - | - | **L** |
| Noazin 2013 | H | H | H | H | NA | L | H | U | L | L | NA | - | - | - | **H** |
| Reyburn 2000 | U | U | H | L | NA | L | L | L | L | L | NA | - | - | - | **L** |
| Rojas 2006 | L | U | H | L | NA | L | L | U | L | L | NA | - | - | - | **L** |
| **Visceral leishmaniasis** | | | | | | | | | | | | | | | |
| Elnaiem 1999 | L | NA | H | L | NA | L | - | - | - | - | - | L | H | L | **L** |
| Joshi 2009 | U | U | H | L | NA | L | - | - | - | - | - | L | L | U | **L** |
| Picado 2010 | L | L | H | L | NA | L | L | L | L | L | NA | L | L | H | **L** |
| **Lymphatic filariasis** | | | | | | | | | | | | | | | |
| Bøgh 1998 | U | U | H | L | NA | L | - | - | - | - | - | L | H | H | **M** |
| Charlwood 1987 | L | NA | H | L | NA | L | - | - | - | - | - | L | H | L | **L** |
| Poopathi 1995 | U | U | H | L | NA | L | - | - | - | - | - | H | H | L | **M** |
| **Dengue** | | | | | | | | | | | | | | | |
| Kroeger 2006 | L | L | H | H | NA | L | - | - | - | - | - | H | L/H* | L | **L** |
| Lenhart 2008 | L | L | H | L | NA | L | - | - | - | - | - | H | L/H* | L | **L** |
| Lenhart 2013 | L | L | H | L | NA | L | - | - | - | - | - | H | L/H* | L | **L** |
| Nguyen 1996 / Igarashi 1997 | U | U | H | L | NA | L | L | U | U | L | NA | H | H | U | **L** |
| Vanlerberghe 2013 | U | U | H | L | NA | L | - | - | - | - | - | H | H | L | **M** |
| **Japanese encephalitis** | | | | | | | | | | | | | | | |
| Dutta 2011 | U | U | H | L | NA | L | L | U | U | L | NA | U | H | U | **L** |
| U = unclear, L = low risk of bias, H = high risk of bias, NA = not applicable, M = medium risk of bias, *used both ovitraps (objective measurement technique, L) and larval surveys (non-objective measurement technique, H) | | | | | | | | | | | | | | | |
