# Supplementary material for: Benefit of Insecticide-Treated Nets, Curtains and Screening on Vector Borne Diseases, Excluding Malaria: A Systematic Review and Meta-analysis
Source: PLoS Negl Trop Dis. 2014 Oct 9;8(10):e3228. doi: 10.1371/journal.pntd.0003228 (PMC4191944; doi:10.1371/journal.pntd.0003228)
Supplement: Supporting Information S9 — Assessment of study quality. (DOCX) [file pntd.0003228.s009.docx]

**Supporting information S9: Assessment of Study Quality**

| Study | Study design | Sample size calculation (overall / clinical) | Sample size calculation (entomological outcomes) | Length of follow up period | Risk of bias | Overall score | Study quality |
| --- | --- | --- | --- | --- | --- | --- | --- |
|  | RCT (+10), crossover / rotational study (+7), pre post study (+4) | Not performed and no sig. effect shown  (-1) | Not performed (-0.5),  <10 sampling sites/ arm (-1) | -0.5 > 1 year/season but limited repeat measures  < 1 year/ transmission season (-1) | Medium (-0.5)  High (-1) |  | ≥ 7 high,  ≥4 < 7 medium,  <4 low |
| **Cutaneous leishmaniasis** | | | | | | | |
| Alexander 1995 | Crossover (+7) | NA | < 10/arm (-1) | < 1 year (-1) | Low (0) | 5 | Medium |
| Alten 2003 | Non-randomised pre post (+4) | Sig. effect shown (0) | Not done (10/arm) (-0.5) | > 1 year (0) | High (-1) | 2.5 | Low |
| Emami 2009 | RCT (+10) | Sig. effect shown (0) | Not done (30 sticky + 20 LT) (-0.5) | 1 year (0) | Low (0) | 9.5 | High |
| Kroeger 2002 | Matched RCT (+10) | Not done and no sig. effect shown (-1) | Not done (565 LT total) (-0.5) | < 1 year (-1)^1^ | Low (0) | 7.5 | High |
| Majori 1989 | Non-randomised pre post (+4)* | NA | < 10/arm (-1) | < 1 year (-1) | High (-1) | 1 | Low |
| Nadim 1995 | RCT (+10) | Not done and no sig. effect shown (-1) | NA | 1 year (0) | Low (0) | 9 | High |
| Noazin 2013 | Non-randomised pre post (+4) | Sig. effect shown (0) | NA | > 1 year (0) | High (-1) | 3 | Low |
| Reyburn 2000 | RCT (+10) | Done (0) | NA | > 1 year | Low (0) | 10 | High |
| Rojas 2006 | RCT (+10) | Sig. effect shown (0) | NA | 1 year (0) | Low (0) | 10 | High |
| **Visceral leishmaniasis** | | | | | | | |
| Elnaiem 1999 | Crossover (+7) | NA | < 10/arm (-1) | < 1 year (-1) | Low (0) | 5 | Medium |
| Joshi 2009 | RCT (+10) | NA | Not done (-0.5) | < 1 year (-1) | Low (0) | 8.5 | High |
| Picado 2010 | RCT (+10) | Done (0) | Not done (≥ 10/arm) (-0.5) | > 1 year (0) | Low (0) | 9.5 | High |
| **Lymphatic filariasis** | | | | | | | |
| Bøgh 1998 | RCT (+10) | NA | Not done (12/arm) (-0.5) | > 1 year but limited repeat measures (-0.5) | Medium (-0.5) | 8.5 | High |
| Charlwood 1987 | Crossover (+7) | NA | < 10/arm (-1) | < 1 year (-1) | Low (0) | 5 | Medium |
| Poopathi 1995 | Non-randomised pre post (+4) | NA | < 10/arm (-1) | < 1 year (-1) | Medium (-0.5) | 1.5 | Low |
| **Dengue** | | | | | | | |
| Kroeger 2006 | RCT (+10) | NA | Not done (> 10/arm) (-0.5) | > 1 year (0) | Low (0) | 9.5 | High |
| Lenhart 2008 | RCT (+10) | NA | Not done (> 10/arm) (-0.5) | > 1 year (0) | Low (0) | 9.5 | High |
| Lenhart 2013 | RCT (+10) | NA | Not done (> 10/arm) (-0.5) | Season (0) | Low (0) | 9.5 | High |
| Nguyen 1996 / Igarashi 1997 | Non-randomised pre post (+4) | Sig. effect shown (0) | Not done (> 10/arm) (-0.5) | Season (0) | Low (0) | 3.5 | Low |
| Vanlerberghe 2013 | RCT (+10) | Done (0) | Not done (>10 arm) (-0.5) | > 1 year but limited repeat measures (-0.5) | Medium  (-0.5) | 8.5 | High |
| **Japanese encephalitis** | | | | | | | |
| Dutta 2011 | Non-randomised pre post (+4) | Sig. effect shown (0) | < 10/arm (-1) | > 1 year (0) | Low (0) | 3 | Low |
| * study is controlled, pre-post crossover design (analysed as if pre-post study), ^1^ follow up period differed for clinical and ento outcomes - was < 1 year for entomological outcomes. | | | | | | | |
